# Aldosterone increases the expression and subcellular localization of SERCA2a and SERCA2b in the rat mesenteric artery

**DOI:** 10.3389/fphys.2026.1811001

**Published:** 2026-05-04

**Authors:** Cristian J. Arriero-Carrillo, Hiram Lozano-Ruiz, Agustín Guerrero-Hernández, Federico Castro-Muñozledo, Angélica Rueda

**Affiliations:** 1 Department of Biochemistry, Center for Research and Advanced Studies (Cinvestav) of the National Polytechnic Institute, Mexico City, Mexico; 2 Department of Cellular Biology, Center for Research and Advanced Studies (Cinvestav) of the National Polytechnic Institute, Mexico City, Mexico

**Keywords:** Aldosterone (ALDO), Ca^2+^ sparks, Ca^2+^ waves, mesenteric artery (MA), SERCA pump, SERCA isoforms, SERCA2a, SERCA2b

## Abstract

Aldosterone (Aldo), a mineralocorticoid hormone, modulates cardiovascular function by regulating the expression of intracellular Ca^2+^ handling proteins, among other effects. In rat resistance-sized mesenteric arteries (MA), Aldo treatment (10 nM, 24 h) upregulates both the L-type voltage-gated Ca^2+^ channel α_1C_ subunit (Ca_V_1.2) and the Sarco/Endoplasmic Reticulum Ca^2+^ ATPase (SERCA pump), thereby increasing Sarcoplasmic Reticulum (SR) Ca^2+^ load. Two SERCA isoforms, SERCA2a and SERCA2b, are expressed in rat MA, but their specific physiological contributions to distinct intracellular Ca^2+^ signals, remain unclear. In this study, we characterized the relative abundance and subcellular distribution of SERCA2a and SERCA2b in rat MA, their regulation by Aldo, and the impact of Aldo-induced SERCA remodeling on local Ca^2+^ signals relevant to vascular function, such as Ca^2+^ sparks and Ca^2+^ waves. Aldo-treated MA smooth muscle cells (MASMC) exhibited increased Ca^2+^ spark frequency and a higher incidence of spontaneous Ca^2+^ waves. Aldo augmented both protein and mRNA levels of SERCA2a and SERCA2b, effects that were blocked by the mineralocorticoid receptor (MR) antagonist RU28318. Under control conditions, SERCA2a was predominantly localized in the perinuclear region, whereas SERCA2b was distributed across both subplasmalemmal and perinuclear regions. Aldo treatment increased the expression of both isoforms in all analyzed subcellular compartments (subplasmalemmal, cytoplasmic, and perinuclear), with a pronounced redistribution towards the subplasmalemmal region of MASMC. This shift in SERCA subcellular distribution likely contributes to enhanced superficial Ca^2+^ buffering and the ignition of Ca^2+^ sparks and Ca^2+^ waves. Furthermore, Aldo increased mRNA levels of mitochondrial transcription factors A and B2 (TFAM and TFB2M), previously implicated in SERCA regulation in human aorta, suggesting a transcriptional mechanism whereby MR activation of the SERCA2 gene is associated with increased TFAM and TFB2M expression. Collectively, these findings demonstrate for the first time that Aldo increases the expression and promotes the subplasmalemmal localization of SERCA2a and SERCA2b in MASMC. This remodeling underscores their critical role in maintaining the superficial Ca^2+^ buffering system and SR Ca^2+^ load to prevent pathological elevations in the intracellular Ca^2+^ concentration. Our results highlight the SERCA pump as a potential therapeutic target in hypertension associated with hyperaldosteronism.

## 1 Introduction

Resistance-sized mesenteric arteries (MA) are part of the gastrointestinal circulation that receives ~25% of total cardiac output under physiological resting conditions. Consequently, vasoconstriction or vaso-relaxation of MA exerts a strong influence on peripheral vascular resistance and systemic blood pressure (Widmaier et al., 2014). Increases in the intracellular Ca^2+^ concentration ([Ca^2+^]_i_) in smooth muscle cells within the MA wall (MASMCs) induces vasoconstriction, whereas a decrease in [Ca^2+^]_i_ promotes their vaso-relaxation (Koenigsberger et al., 2004; Tykocki et al., 2017; Brown et al., 2018; Jackson, 2021). Thus, intracellular Ca^2+^ signals in MASMCs function as master regulators of vascular function. Elevations in intravascular pressure activate L-type voltage-gated Ca^2+^ channels (LTCC), generating distinctive intracellular Ca^2+^ signals in vascular smooth muscle cells (VSMC), such as Ca^2+^ sparks and Ca^2+^ waves. These signals arise through mechanisms involving SR Ca^2+^ recapture via the Sarco/Endoplasmic Reticulum Ca^2+^ ATPase (SERCA pump) (Jaggar, 2001; Hill-Eubanks et al., 2011). Ca^2+^ sparks are localized, transient increases in [Ca^2+^]_i_ produced by the activation of clusters of Ca^2+^ channels/Ryanodine receptors (RyRs) in the sarcoplasmic reticulum (SR) (Nelson et al., 1995; Cheng and Lederer, 2008). Ca^2+^ sparks typically occur near the plasma membrane (PM), where they activate large-conductance Ca^2+^-activated K^+^ channels (K_Ca_1.1) that generate spontaneous transient outward currents which causes PM hyperpolarization. As a result, the open probability (*Po*) of LTCC decreases, which reduces [Ca^2+^]_i_ promoting vasorelaxation (Nelson et al., 1995; Jaggar et al., 1998; Cheranov and Jaggar, 2002; Heppner et al., 2002; Rueda et al., 2013). Ca^2+^ waves, in contrast, are intracellular Ca^2+^ elevations that propagate throughout the cytoplasm of VSMCs. Their function is not yet fully understood. Some studies suggest they participate in vasorelaxation (Jaggar, 2001), while others report that Ca^2+^ waves contribute to contraction (Lamont and Wier, 2004; Amberg and Navedo, 2013). This complex Ca^2+^ signaling is highly dependent on SR Ca^2+^ load, which is maintained by the SERCA pump, responsible for recapturing Ca^2+^ into the SR, and contributing to the generation of local Ca^2+^ signals (Wray and Burdyga, 2010; Takeda et al., 2011; Fransen et al., 2020).

MASMCs mainly express the SERCA isoforms SERCA2a and SERCA2b (Lagaud et al., 1999). SERCA2b is the predominant isoform in several types of blood vessels (Fransen et al., 2020); however, this observation has not been confirmed for MA yet. The importance of determining the subcellular localization of the SERCA isoforms has been evidenced by the demonstration of functional SR compartmentalization in VSMCs, suggesting that the stimulus-dependent Ca^2+^ mobilization is linked to the differential subcellular distribution of the SERCA isoforms (Van Breemen et al., 1995; Clark et al., 2010). In pulmonary artery VSMCs, SERCA2a and SERCA2b exhibit differential subcellular distribution, SERCA2a is predominantly localized to the perinuclear region (~90%), whereas SERCA2b is enriched in the superficial SR near the PM (~72%). This subplasmalemmal region is also known as PM-SR nanodomain (Clark et al., 2010). Such subcellular distribution supports the hypothesis that SERCA2 isoforms serve distinct SR Ca^2+^ compartments with specialized physiological function (Clark et al., 2010).

The transcriptional regulation of SERCA2 gene has been extensively studied revealing multiple mechanisms that control its expression (Watanabe et al., 2011; Zarain-Herzberg et al., 2012, Zarain-Herzberg et al., 2014). The SERCA2 gene contains a promoter region with binding sites for several transcription factors (Watanabe et al., 2011; Zarain-Herzberg et al., 2012). It was shown that both mitochondrial transcription factors A and B2 (TFAM and TFB2M) regulate SERCA2 expression through direct binding to its promoter (Watanabe et al., 2011). Aldosterone (Aldo) stimulation decreases the expression of these transcription factors in human aortic smooth muscle cells (HAOSMCs) (Chou et al., 2015); however, their transcriptional effects on SERCA2 gene in rat MAs have not been studied.

Aldo, a mineralocorticoid hormone, exerts its effects by interacting with the mineralocorticoid receptor (MR), and plays a central role in cardiovascular function and blood pressure (BP) regulation (Lother et al., 2015). Among its actions, Aldo modulates the transcription of proteins involved in intracellular Ca^2+^ handling. For instance, Aldo increases the expression of the LTCC α_1C_ subunit (Ca_V_1.2) in the heart, aorta, coronary arteries, and MA (Lalevée et al., 2005; Mesquita et al., 2018; Salazar-Enciso et al., 2022). Evidence also suggests that transcriptionally regulates SERCA2 pump expression in aorta and MA (Chou et al., 2015; Salazar-Enciso et al., 2022).

Our previous work demonstrated that rat MA treated with Aldo (10 nM, 24h) exhibit increased Ca_V_1.2 expression. Despite this, depolarization-induced vasoconstriction remains unchanged. This outcome was partially attributed to the augmented SR Ca^2+^ load in Aldo-treated MASMC resulting from increased expression and activity of SERCA2 (Salazar-Enciso et al., 2022). However, it is unclear whether this effect was due to selective upregulation of the predominant isoform SERCA2b or involves both major isoforms in MASMC. In addition, whether Aldo induces remodeling in SERCA subcellular distribution potentially accounting for alterations in intracellular Ca^2+^ dynamics of MASMCs remains unknown.

In this work, we studied the effects of Aldo, a key regulator of vascular physiology, in the expression and subcellular redistribution of SERCA2 pump isoforms in MASMC. After characterizing the isoform-specific expression, subcellular distribution, and Aldo-driven transcription of SERCA2a and SERCA2b in rat MA, we reveal novel mechanisms linking mineralocorticoid effects to local Ca^2+^ signals such as Ca^2+^ sparks and Ca^2+^ waves in MASMCs. These findings highlight the physiological relevance of SERCA2 remodeling in vascular function and its potential as a therapeutic target in Aldo-driven hypertension.

## 2 Material and methods

All procedures were performed according to the ethical guidelines of the Mexican Official Norm (NOM-062-ZOO-1999) and the National Institutes of Health Guide for the Care and Use of Laboratory Animals (NIH publication updated in 2011). The animal protocol was approved by the Institutional Bioethical Committee for Care and Handling of Laboratory Animals at the Cinvestav (CICUAL Protocol No. 0100-14). Unless specified in **Supplementary Table 1**, all reagents were obtained from Merck Mexico, a subsidiary of Merck KGaA, Darmstadt, Germany.

### 2.1 Aldosterone treatment of rat mesenteric arteries

Dissection of MAs and treatment with Aldo were carried out according to a previously reported protocol (Salazar-Enciso et al., 2022). Briefly, twelve-week-old male Wistar rats (250–300 g body weight) were anesthetized with sodium pentobarbital (100 mg/kg of body weight, intraperitoneally). The mesentery was excised from the peritoneal cavity and placed in a Petri dish with ice-cold HEPES-buffered dissection solution (composition in mM: 80 glutamic acid, 80 NaOH, 55 NaCl, 6 KCl, 2 MgCl_2_, 10 D-glucose, 10 HEPES, pH 7.4 with NaOH). Third and four order branches of resistance-sized MAs were isolated under the microscope and carefully cleaned of fat, connective tissue, and blood. The isolated arteries were then transferred to serum-free Dulbecco’s Modified Eagle’s Medium (DMEM) supplemented with 100 U/mL penicillin and 100 µg/mL streptomycin. MA segments were cultured in a humidified atmosphere of 5% CO_2_ in the presence of 10 nM Aldo, 1 µM RU28318 + 10 nM Aldo or in its absence (control group). After 24-h incubation, MA segments were either loaded with the Ca^2+^ indicator Fluo 4-AM for recordings of Ca^2+^ sparks and Ca^2+^ waves in single MASMC within the arterial wall (by confocal microscopy in the *line-scan* mode), or frozen at –72 °C for subsequent Western blot and real-time qPCR analyses. Only for immunocytochemistry experiments, MA segments were subjected to controlled enzymatic digestion to isolate single MASMCs.

### 2.2 Recordings of Ca^2+^ sparks and Ca^2+^ waves in MASMCs

Resistance-sized MA were loaded with the Ca^2+^ indicator Fluo 4-AM (10 µM for 40 min) in high potassium physiological saline (20 mM, PSS-20K). MA segments were placed on a glass coverslip attached to a perfusion chamber with PSS, allowed to adhere, and perfused with PSS-20K at room temperature (RT). Ca^2+^ sparks and Ca^2+^ waves were recorded in individual MASMCs within the arterial wall using the *line-scan* mode of a laser scanning confocal microscope (Zeiss, LSM 900, Carl Zeiss of México S.A. de C.V.), the recordings were performed with a 63x oil immersion objective, in *line-scan* mode, 10 images per cell of 1000 lines each, at a speed of 3.05 ms/line. The images were analyzed with the Interactive Data Language (IDL) software (version 5.5, Research System Inc.) using the home-made protocol developed by Ana Maria Gómez Ph.D., FESC, FISHR. (Inserm UMR-S 1180, Université Paris-Saclay, Orsay, France) (Gómez et al., 2009). Images of Ca^2+^ sparks were normalized by dividing the fluorescence intensity of each pixel (F) by the average basal fluorescence intensity (F_0_) within the cell to generate a normalized image (F/F_0_). The Ca^2+^ spark frequency was expressed as the number of events recorded per cell in 10 scans per line (events/s) as previously reported (Rueda et al., 2013). The percentage of cells with Ca^2+^ waves was determined by analyzing the records of 10 images obtained per cell in *line-scan* mode with Zeiss ZEN 2010 software.

### 2.3 Western blot

Protein expression of SERCA2 isoforms in MA was determined by Western blot, as previously reported (Salazar-Enciso et al., 2022) with some modifications described as follows. After 24 h-treatment in the presence or in the absence of Aldo, pools of resistance-sized MA from 4 rats were pulverized in liquid N_2_ and homogenized in 300 µL of ice-cold homogenization buffer (composition in mM: 20 HEPES, 20 NaF, 300 sucrose, 0.5% sodium deoxycholate, pH 7.2 with NaOH) supplemented with protease inhibitor cocktail (1 µg/mL aprotinin, 500 µM benzamidine, 12 µM leupeptin, 100 µM PMSF), centrifuged at 2000 x *g* for 10 min at 4 °C and the supernatant was collected. Protein concentration was determined by the bicinchoninic acid (BCA) method. Samples were separated in a 4, 8 and 12% discontinuous gradient SDS-PAGE (20 µg of protein per well) for 1 h at 90 V and 1 h 20 min at 100 V and transferred to PVDF membranes for 2 h, 100 V at 4 °C and blocked with 5% blotto in Tris-buffered saline solution with 0.1% Tween 20 (TBS-T) for 1 h, subsequently the PVDF membranes were incubated with primary antibodies (**Supplementary Table 2**) for 2 h at RT. The specificity of SERCA antibodies against each SERCA isoform was assessed using homogenates from different cells or tissues enriched with SERCA2a (cardiomyocytes) or SERCA2b (CHO-K1 cells) (*Data not shown*). The PVDF membranes were then incubated with horseradish peroxidase-conjugated anti-rabbit or anti-mouse secondary antibodies. The protein band was detected by chemiluminescence on X-ray film. Protein bands were scanned and densitometric quantification was performed using ImageJ/Fiji software (v.1.54p, National Institutes of Health, USA). GAPDH was used as a loading control.

### 2.4 Total RNA extraction and real-time quantitative PCR

Total RNA was extracted from MA pooled from 3–4 rats with TRIzol Reagent^®^ following a previously reported protocol (Salazar-Enciso et al., 2022). We obtained a total mRNA yield of 661.0 ± 34.75 ng/µL in control arteries (n = 4 independent experiments), and 715.9 ± 46.81 ng/µL in Aldo-treated arteries (n = 4 independent experiments). Total RNA was treated with DNAse for 20 min at 37 °C, and mRNA integrity was determined by agarose gel electrophoresis. The reverse transcription was carried out with the Superscript II Reverse Transcriptase Kit (Thermo Fisher Scientific, Inc., Waltham, MA USA) according to the manufacturer’s instructions. The cDNA samples were amplified by real-time qPCR using the QuantiNova SYBR Green PCR kit. **Supplementary Table 3** lists the specific primers for each gene of interest. *Gapdh* was used as a reference gene. All samples were run in triplicate. Differences in expression were determined using the 2ΔΔCT method (Livak and Schmittgen, 2001).

### 2.5 Mesenteric artery smooth muscle cell isolation

MASMCs were isolated enzymatically following a previously described protocol (Salazar-Enciso et al., 2022) with some modifications as described below. The segments of MAs were transferred into an Eppendorf microtube containing 1 mL of dissection solution containing (in mg/mL: 1 papain, 1 bovine serum albumin, 1 dithiothreitol). MAs were incubated at 37 °C for 15 min. The papain-treated arteries were then transferred to 1 mL of dissection solution containing (in mg/mL) 1.43 collagenase F, 1 collagenase H, 0.011 CaCl_2_ and incubated for 8 min at 37 °C. The digestion was stopped by washing the tissue three times, 2 min each, with dissection solution. Cells were mechanically dissociated from the vessels using a transfer pipette. The cells suspension was used on the same day.

### 2.6 Immunocytochemistry

Isolated MASMCs were plated on circular coverslips and allowed to adhere for 10 min, fixed with 4% paraformaldehyde for 20 min, washed three times with phosphate buffered saline (PBS), permeabilized, and blocked with 1 mL of 5% (v/v) sheep serum in PBS/Triton 0.2% (v/v; PBS-T) for 30 min, at RT, as previously described (Rueda et al., 2006). Then, myocytes were washed three times with PBS and incubate overnight whit sequence-specific antibodies for SERCA2a and SERCA2b (1:200), diluted in PBS-T with 1% sheep serum. MASMCs were washed three times with PBS and incubated with Alexa-Fluor 488 fluorophore-conjugated anti rabbit secondary antibody (1:400, Invitrogen Cat# A31627) for 2 h at RT in the dark, washed three times with PBS. Coverslips were mounted on microscope slide with mounting solution containing DAPI (SlowFade™ Diamond Antifade Mountant with DAPI, **Supplementary Table 1**). Images were taken with a Zeiss LSM 900 confocal microscope using Zen 210 software (Carl Zeiss de México S.A. de C.V.). Cells were imaged with a Zeiss Apochromatic 63x oil immersion objective; the pinhole was optimized to 1 Airy unit (pixel size 0.071 µm x 0.071 µm x 0.240 µm).

Image processing and immunofluorescence quantifications were performed using the Image J/Fiji software (v.1.54p, National Institutes of Health, USA). From each image, the entire cellular area or specific regions of cell were selected, and the fluorescence corresponding to the pixels included in each selection was obtained. The background fluorescence (F_0_) was obtained outside of the cell area and was subtracted from the total fluorescence of each cell. Following previous work (Clark et al., 2010; Vaithianathan et al., 2010) we delimited three subcellular regions of interest (subplasmalemmal, cytoplasmic and perinuclear) in the MASMC and analyzed the subcellular distribution of SERCA2 isoforms. Each subcellular region was delimited with the regions of interest (ROIs) tool, and the corresponding immunofluorescence from each SERCA2 isoform was quantified.

### 2.7 Statistical analysis

Data are presented as the mean ± standard error of the mean (M ± S.E.M.). Statistical analysis was performed using GraphPad Prism 8.0.1 (GraphPad Software, Inc.). The Shapiro-Wilk test was used to analyze the normal distribution of the data. Statistical significance was determined by Student’s t-test or one-way analysis of variance (ANOVA, ordinary or repeated means) followed by Tukey’s *post-hoc* test; when data failed the normality distribution test, statistical significance was determined by Mann-Whitney rank sum test or Kruskal-Wallis one-way ANOVA followed by Dunn’s *post-hoc test*. A *P* value < 0.05 was considered statistically significant.

## 3 Results

### 3.1 Aldosterone increases Ca^2+^ spark frequency and spontaneous Ca^2+^ wave incidence in mesenteric artery smooth muscle cells

**Figure 1:**
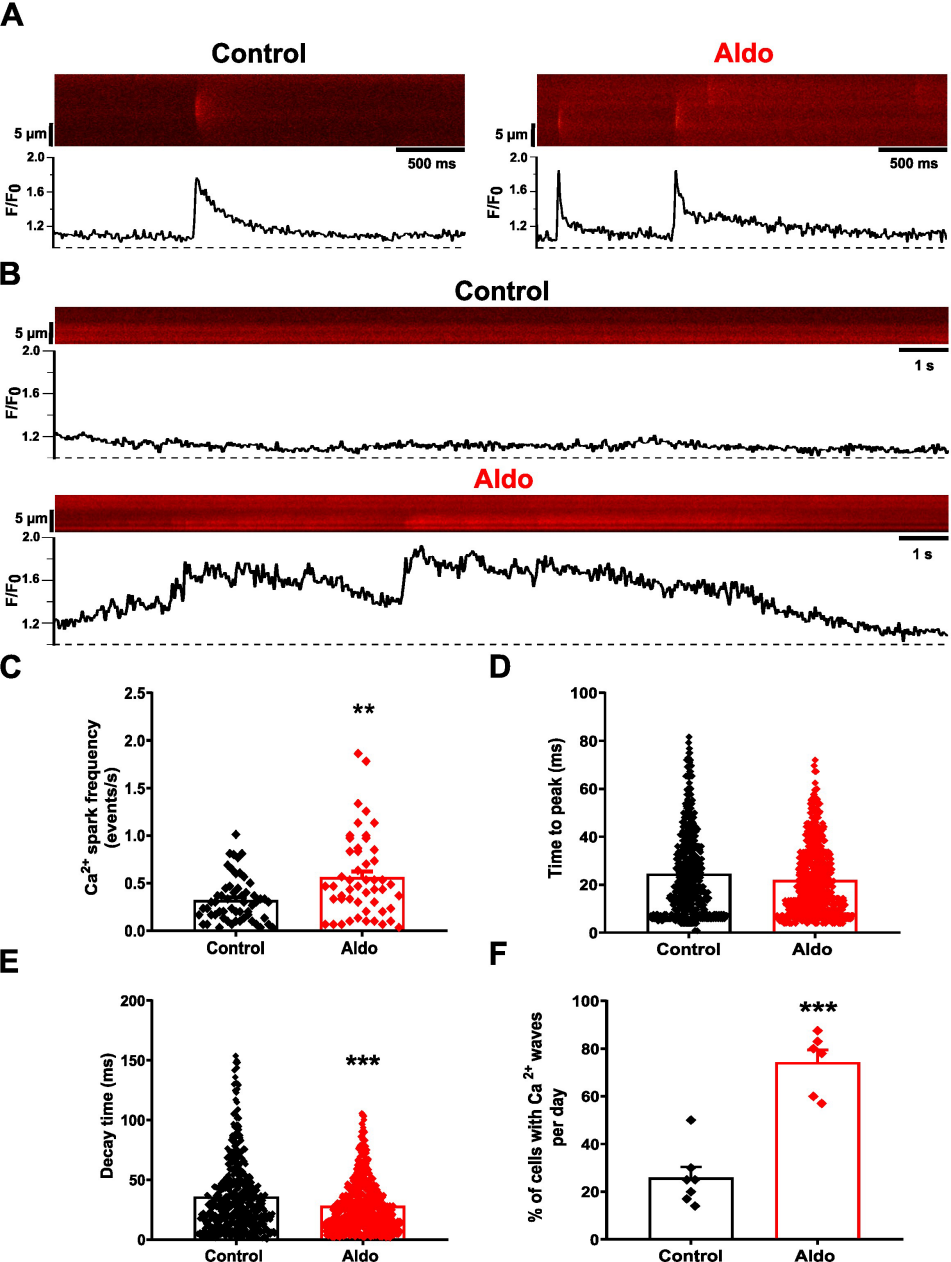
Aldosterone treatment increases Ca^2+^ spark frequency and spontaneous Ca^2+^ waves in rat MASMC. **(A)** Representative pseudo-colored confocal images of Ca^2+^ sparks (*top*) and normalized (F/F_0_) fluorescence profiles (*bottom*) of Fluo 4-loaded control and Aldo-treated MASMC (10 nM, 24 h). Ca^2+^ sparks were recorded in *line-scan* mode, 1000 lines each image, at 3.05 ms/line. **(B)** Representative pseudo-colored confocal images of spontaneous Ca^2+^ waves (*top*) and normalized (F/F_0_) fluorescence profiles (*bottom*) in control and Aldo-treated MASMC. Ca^2+^ waves were recorded in the *line scan* mode, 8000 lines each image, at 3.05 ms/line. **(C–E)**. Scatter plots with bar graphs showing Ca^2+^ spark frequency (**(C)**, determined as the number of Ca^2+^ events observed per s), Ca^2+^ spark time-to-peak (**(D)**, in ms), and Ca^2+^ spark decay time (**(E)**, in ms). Data are shown for control (*black symbols*/*bars*, 469 sparks, n = 57 cells/N = 7 rats) and Aldosterone-treated MASMC (*red symbols*/*bars*, 630 sparks, n = 50 cells/N = 6 rats). Values are presented as M ± S.E.M. The Mann-Whitney U test was used to determine significant differences; **P ≤ 0.01, ***P ≤ 0.001 *vs*. control group. **(F)** Scatter plot with bar graph of Ca^2+^ wave incidence in control (*black symbols/bar*, n = 7 recording days/N = 7 rats) and Aldosterone-treated MASMC (*red symbols*/*bar*, n = 6 recording days/N = 6 rats). Values are presented as M ± S.E.M. Statistical analysis: unpaired Student´s *t* test. *** *P* ≤ 0.001 *vs*. control group.

Ca^2+^ sparks are localized Ca^2+^ release events that promote vasorelaxation. In cerebral arteries, their frequency is tightly controlled by the SR Ca^2+^ load (Cheranov and Jaggar, 2002). We have previously reported that short-term Aldo treatment (10 nM, 24 h) increases SR Ca^2+^ load in MASMCs due to augmented Ca^2+^ influx (Salazar-Enciso et al., 2022). Therefore, we analyzed the frequency of Ca^2+^ sparks and the incidence of spontaneous Ca^2+^ waves in Aldo-treated MASMCs. Using the *line-scan* mode of the confocal microscope, we selectively analyzed individual MASMCs within the arterial wall, enabling quantification of Ca^2+^ sparks and Ca^2+^ waves at the single-cell level in Fluo 4-loaded MA segments. **Figure 1A** shows representative two-dimensional confocal images of Ca^2+^ sparks with respective normalized fluorescence profiles (F/F_0_), recorded in individual MASMCs of Fluo 4-loaded MAs treated or not (control) with Aldo (10 nM, 24 h). We found that the Ca^2+^ spark frequency was significantly increased in Aldo-treated MASMCs compared with control cells (**Figure 1C**). When analyzing kinetic characteristics such as Ca^2+^ spark time-to-peak (TTP), an indicator of Ca^2+^ release speed during the rising phase of the Ca^2+^ spark (Cheng and Lederer, 2008), this parameter remained unchanged after Aldo treatment (**Figure 1D**). In contrast, the Ca^2+^ spark decay-time, a parameter linked to cytoplasmic Ca^2+^ clearance mechanisms, was significantly decreased (**Figure 1E**), suggesting an important contribution of the SERCA pump in local Ca^2+^ decline of the Ca^2+^ spark.

Because the initiation of spontaneous Ca^2+^ waves can also be influenced by the SR Ca^2+^ load, we also analyzed the incidence of Ca^2+^ waves. **Figure 1B** shows representative two-dimensional confocal images of Ca^2+^ waves and their respective normalized (F/F_0_) fluorescence profiles, recorded in Fluo 4-loaded MASMCs treated or not (control) with Aldo (10 nM, 24 h). The Ca^2+^ wave incidence was significantly augmented in Aldo-treated MASMC (**Figure 1F**). These results support the idea that Aldo modifies the intracellular Ca^2+^ dynamics of MASMCs due to an increased SR Ca^2+^ load (Salazar-Enciso et al., 2022), and highlight the importance of the SERCA pump in maintaining Ca^2+^ spark and wave activity and in buffering the enhanced Ca^2+^ influx in Aldo-treated MASMCs.

The essential role of SERCA in maintaining SR Ca^2+^ load and enabling Ca^2+^ spark/wave activity has been demonstrated using SERCA inhibitors, such as thapsigargin (TGN) or cyclopiazonic acid (CPA). In myogenic cerebral arteries, TGN (100 nM) rapidly depleted SR Ca^2+^ content, abolishing Ca^2+^ sparks/waves (Jaggar, 2001). At lower concentration (20 nM) TGN progressively reduced Ca^2+^ spark frequency, amplitude, and spatial spread, but not decay time (Cheranov and Jaggar, 2002). To confirm the crucial role of SERCA for the generation of Ca^2+^ sparks/waves in MASMC, we recorded Ca^2+^ spark/waves before and after incubation with CPA (10 µM, 10 min). SERCA inhibition abolished both Ca^2+^ spark activity (**Supplementary Figure 1**) and Ca^2+^ wave incidence (*data not shown*) in control and Aldo-treated cells, underscoring the essential role of SERCA in sustaining Ca^2+^ spark/wave activity.

### 3.2 Aldosterone increases the expression of both SERCA2 pump isoforms in rat mesenteric artery

**Figure 2:**
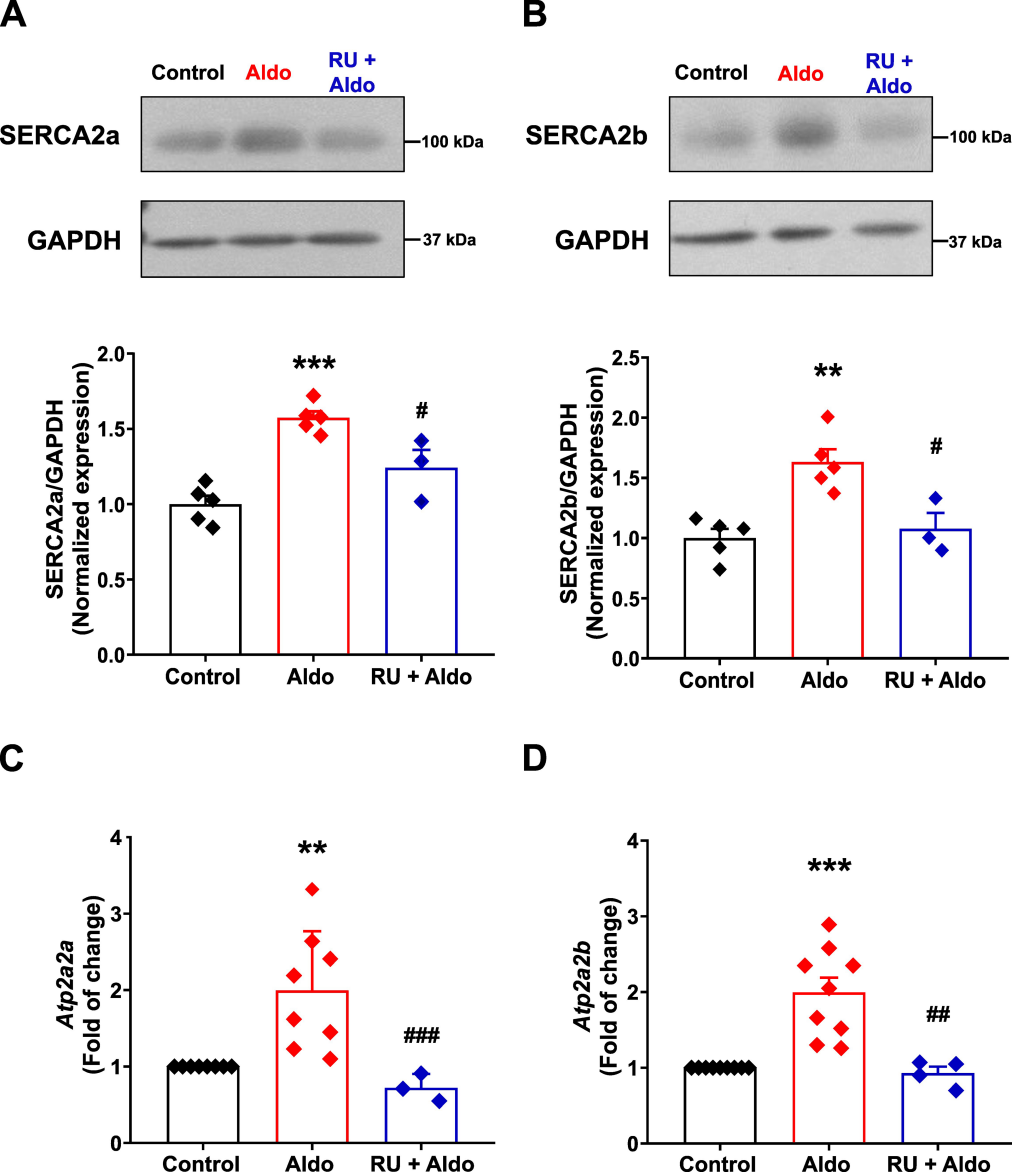
Aldosterone treatment increases protein expression and gene transcription of SERCA2a and SERCA2b in rat MA. **(A, B)**. Representative immunoblots (*top*) and corresponding normalized scatter plots with bar graphs (*bottom*) showing protein levels of SERCA2a **(A)** and SERCA2b **(B)** in control (*black symbols/bars*; n = 5 independent experiments/N = 18 rats), Aldosterone-treated (10 nM; *red symbols/bars*; n = 5 independent experiments/N = 18 rats) and RU28318 (1 µM) + Aldosterone (10 nM) co-treatment (*blue symbols/bars*; n = 3 independent experiments, N = 12 rats) MAs. GAPDH was used as loading control. Data are presented as M ± S.E.M. Statistical analysis: One-way ANOVA followed by Tukey *post-hoc* test. **P ≤ 0.01, ***P ≤ 0.001 *vs*. control group, ^#^*P* ≤ 0.05 *vs*. Aldosterone-treated group. Each independent experiment was performed using pooled MA from 3–4 animals. **(C, D)**. Scatter plots with bar graphs showing relative mRNA levels of SERCA2a **(C)** determined by real-time qPCR in control (*black symbols/bars*; n = 8 independent experiments/N = 27 rats), Aldosterone-treated (10 nM; *red symbols/bars*; n = 8 independent experiments/N = 27 rats) and RU28318 (1 µM) + Aldosterone (10 nM) co-treated (*blue symbols/bars*; n = 3 independent experiments/N = 12 rats) MAs; and SERCA2b **(D)**, determined by real-time qPCR in control (*black symbols/bars*; n = 9 independent experiments/N = 31 rats), Aldosterone-treated (10 nM; *red symbols/bars*; n = 9 independent experiments/N = 31 rats) and RU28318 (1 µM) + Aldosterone (10 nM) co-treated (*blue symbols/bars*; n = 4 independent experiments/N = 16 rats) MA. GAPDH was used as reference gene. Data are presented as M ± S.E.M. Statistical analysis: Kruskal-Wallis test followed by Dunn´s multiple comparisons test. **P ≤ 0.01, ***P ≤ 0.001 *vs.* control group. ^##^*P* ≤ 0.01, ^###^*P* ≤ 0.001, *vs*. Aldosterone-treated group.

In MASMC, SR Ca^2+^ load is regulated by the SERCA pump activity, with SERCA2a and SERCA2b as the predominant isoforms in the vasculature (Fransen et al., 2020). We have previously demonstrated that short-term Aldo treatment of MA increases the expression of the generic SERCA2 pump (Salazar-Enciso et al., 2022); however, it remains unclear whether the transcriptional effects of Aldo selectively upregulates one or both isoforms. Therefore, we determined the expression levels of both isoforms by Western blot analysis using specific primary antibodies against each SERCA isoform (**Supplementary Table 2**). Our results show that SERCA2a (**Figure 2A**) and SERCA2b (**Figure 2B**) protein levels were significantly increased in Aldo-treated MAs with respect to control arteries, and this effect was reversed by the co-treatment with the selective MR antagonist, RU28318 (**Figures 2A, B**). Given that Aldo increased the protein expression of both SERCA isoforms, we analyzed the effect of the mineralocorticoid on the transcriptional levels. By semi-quantitative real-time qPCR analysis, we measured mRNA levels using SERCA isoform-specific primers (**Supplementary Table 3**). Firstly, we validated our reference gene (*Gapdh*), observing that its amplification cycle (C_t_) did not change in Aldo-treated MAs compared to the control condition (**Supplementary Figure 2**). We also assessed the relative abundance of each SERCA isoform in control MA. The SERCA2a isoform was expressed at a lower percentage (28.7 ± 1.98%) compared to SERCA2b (71.3 ± 1.98%), under control conditions. The treatment with Aldo increased by 2-fold the mRNA levels of both SERCA2a (**Figure 2C**) and SERCA2b (**Figure 2D**) isoforms. The Aldo-induced increase in mRNA levels of both SERCA isoforms were blocked by the MR antagonist, RU28318, demonstrating the specificity of Aldo modulation of the SERCA pump. Our results indicate that Aldo activates the MR receptor and increases SERCA2a and SERCA2b by transcriptionally regulating SERCA gene.

As previously reported, Aldo induces phenotypic changes in VSMC, promoting the transition from a contractile to a proliferative state (Pruthi et al., 2014). Phenotypic transitions of VSMC have been directly correlated with changes in SERCA pump expression (Berra-Romani et al., 2008; Bobe et al., 2010). To determine that the Aldo-induced increase in SERCA2a and SERCA2b expression under our experimental conditions was not attributable to phenotypic transitions, we assessed the thickness of the tunica media and measured mRNA levels of transgelin (also known as SM22, a specific marker of VSMC phenotypic state). Our results showed that short-term Aldo treatment did not alter tunica media thickness in MA segments, neither affect transgelin mRNA levels compared with controls (**Supplementary Figure 3**). Therefore, the Aldo-induced upregulation of SERCA2 isoforms is not associated with phenotypic changes in rat MA.

### 3.3 SERCA2a and SERCA2b are differentially distributed in mesenteric artery smooth muscle cells

SERCA2a and SERCA2b are the predominant isoforms of the SERCA pump in several vascular beds. In pulmonary artery VSMC, SERCA2a is primarily distributed in the perinuclear region, whereas SERCA2b is enriched in the subplasmalemmal region (Clark et al., 2010).Their distinct subcellular localization suggests that they contribute differently to vascular physiology.

**Figure 3:**
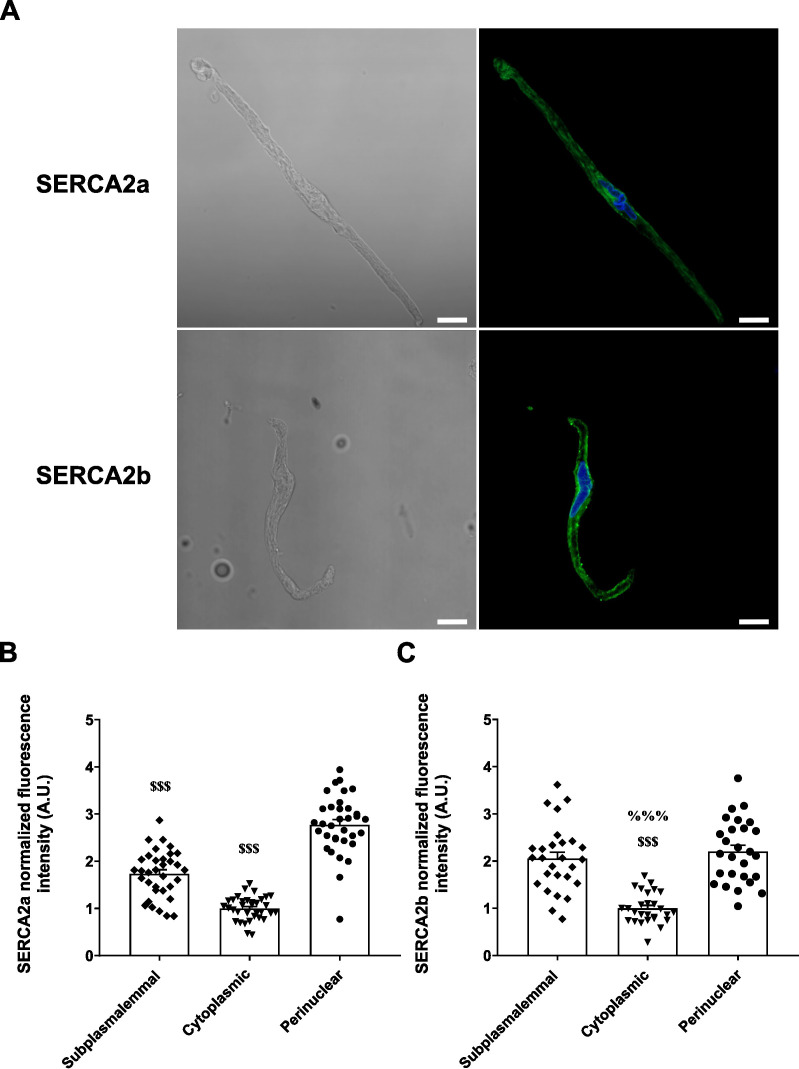
Differential subcellular distribution of SERCA2a and SERCA2b in isolated rat MASMC. **(A)** Representative transmittance images (*left*) and corresponding fluorescence immunostainings (*right*) for SERCA2a (*top*) and SERCA2b (*bottom*) isoforms, visualized by confocal microscopy. Isolated rat MASMCs were immunostained with isoform-specific antibodies (SERCA2a or SERCA2b) and detected using Alexa Fluor 488-conjugated goat anti-rabbit IgG (*green*). Fluorescence intensity was subdivided into three distinct subcellular regions and quantified as described in Material and Methods. Nuclei were stained with DAPI (*blue*). Scale bar = 10 µm. **(B, C)**. Scatterplots with bar graphs showing normalized fluorescence intensity (AU) for SERCA2a **(B)** and SERCA2b **(C)** across subplasmalemmal (*black diamonds*), cytoplasmic (*black triangles*) and perinuclear (*black circles*) regions. SERCA2a immunostainings: n = 34 cells/N = 6 rats. SERCA2b immunostainings: n = 27 cells/N = 6 rats. Data are presented as M ± S.E.M. Statistical analysis: one-way ANOVA followed by Tukey´s *post hoc* test. ^$$$^*P* ≤ 0.001 *vs.* perinuclear region; ^%%%^*P* ≤ 0.001 *vs*. subplasmalemmal region.

Confocal images of control MASMC immunostained with SERCA2a and SERCA2b sequence-specific antibodies (**Supplementary Table 2**) revealed distinct subcellular distributions of SERCA isoforms (**Figure 3A**). The analysis of immunostainings showed that SERCA2a was predominantly localized to the perinuclear region of the cells, where its expression was significantly higher compared to the cytoplasmic or subplasmalemmal compartments (**Figure 3B**). In contrast, SERCA2b was found at both the subplasmalemmal and perinuclear regions, with no significant difference between them, while showed markedly lower expression in the cytoplasmic region (**Figure 3C**). These findings suggest that in rat MASMCs, SERCA2a is functionally restricted to the SR domains close to the nucleus, maintaining SR Ca^2+^ stores that provide Ca^2+^ for contraction or gene transcription in MASMC; whereas SERCA2b, the predominant isoform in MASMCs, is mainly positioned at the superficial SR, where the PM-SR nanodomains are formed to buffer Ca^2+^ influx.

### 3.4 Aldosterone increases the expression of SERCA2a and SERCA2b in all subcellular regions in mesenteric artery smooth muscle cells

**Figure 4:**
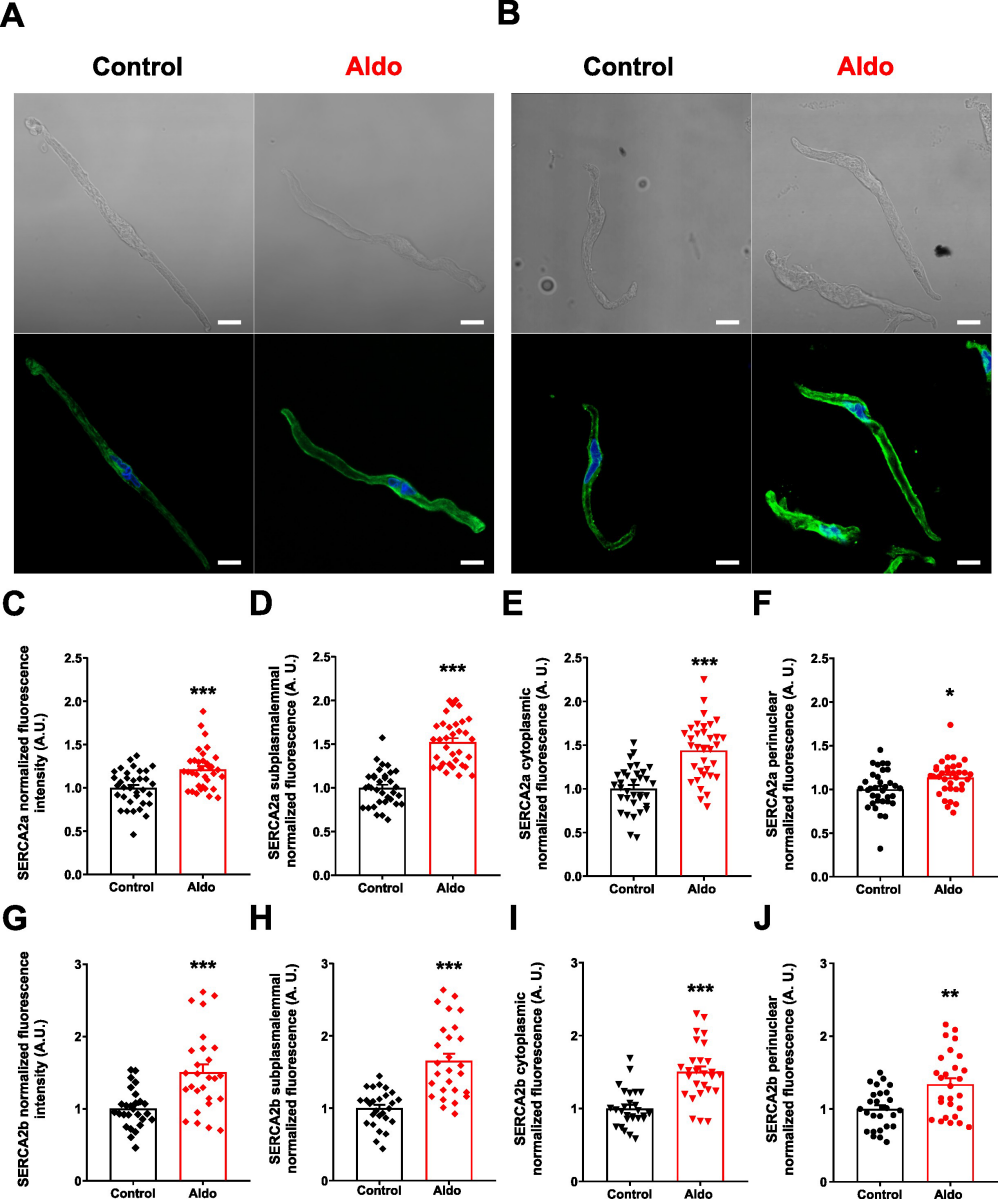
Aldosterone treatment increases protein expression and subcellular localization of SERCA2a and SERCA2b in all subcellular regions in MASMC. Representative transmittance (*top*) and corresponding fluorescence immunostaining (*bottom*) for SERCA2a **(A)** and SERCA2b **(B)** subcellular distribution in control (*left image*) and Aldosterone-treated (10 nM, 24 h; *right image*) MASMCs. Scale bar = 10 µm. **(C–J)**. Scatter plots with bar graphs of fluorescence intensity for SERCA2a at the total cell area **(C)**, subplasmalemmal **(D)**, cytoplasmatic **(E)** and perinuclear **(F)** localizations. For SERCA2b at the total cell area **(G)**, subplasmalemmal **(H)**, cytoplasmatic **(I)** and perinuclear **(J)** regions, in control (*black symbols/bar*) and Aldosterone-treated (*red symbols/bar*) MASMCs. SERCA2a, n = 34 cells/N = 6 rats for both experimental conditions, and SERCA2b, n = 27 cells/N = 6 rats for both experimental conditions. Data are presented as M ± SEM. Statistical analysis: Unpaired Student t-test. *P ≤ 0.05, **P ≤ 0.01 and ***P ≤ 0.001 *vs.* control group.

Beyond changes in overall expression, it has been proposed that the spatial distribution of SERCA2 within specific subcellular regions can modulate intracellular Ca^2+^ signaling. Both thyroid hormones and glucose at supraphysiological levels modulate the expression and subcellular localization of the SERCA pump (Arruda et al., 2005; Searls et al., 2010). Thus, we examined the changes in the Aldo-induced expression of SERCA2a and SERCA2b in subcellular regions of MASMCs. Confocal images of Aldo-treated MASMCs labeled with SERCA2a or SERCA2b specific antibodies and their respective controls (in the absence of the mineralocorticoid) were obtained (**Figures 4A, B**). The images were analyzed by quantifying the absolute fluorescence across the total cell area. The results showed an increased fluorescence for both SERCA2a (**Figure 4C**) and SERCA2b (**Figure 4G**) in Aldo-treated MASMCs. This increase in the total fluorescence of each SERCA isoform correlates directly with the augmented expression of both isoforms, demonstrated by the Western blot and RT-qPCR assays (**Figure 2**). To assess the effects of Aldo across distinct subcellular region (subplasmalemmal, cytoplasmic, and perinuclear), we quantified absolute fluorescence within each cell compartment. This analysis aimed to confirm whether the phenomenon observed in the total fluorescence of each isoform was present across all regions. In control MASMCs, SERCA2a was primarily localized to the perinuclear region. Following Aldo treatment, its expression significantly increased across all subcellular regions, with a more pronounced elevation in the subplasmalemmal region compared with the perinuclear compartment (**Figures 4D–F**). Similarly, SERCA2b was primarily expressed in the perinuclear and subplasmalemmal regions of control MASMCs. Aldo-treatment increased the absolute fluorescence across all subcellular regions (**Figures 4H–J**), indicating that Aldo increased the expression of both isoforms in all three subcellular regions analyzed.

### 3.5 Aldosterone induces the remodeling of SERCA2a and SERCA2b subcellular distributions in MASMCs

**Figure 5:**
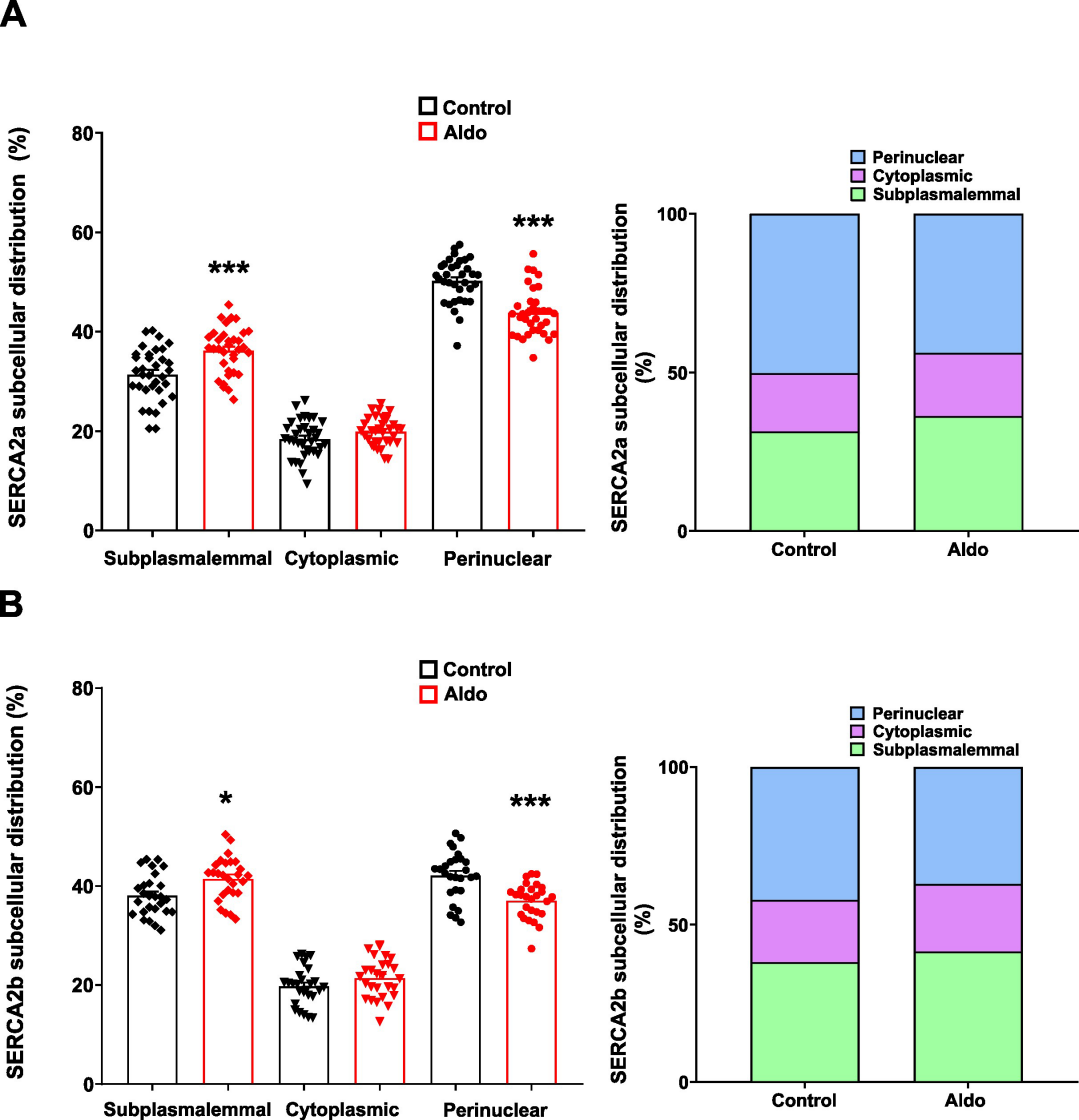
Aldosterone treatment modifies the subcellular distribution of SERCA2a and SERCA2b in MASMCs. **(A, B)**. Scatter plots with bar graph (*left*) and stacked bar graphs (*right*) showing the subcellular distribution of SERCA2a **(A)** and SERCA2b **(B)** at each of the subcellular regions of MASMC from non-treated (*black symbols/bar*) or treated with Aldo (10 nM, 24 h; *red symbols/bar*). SERCA2a: n = 34 cells/N = 6 rats; SERCA2b: n = 27 cells for each condition/N = 6 rats. Data are presented as the M ± SEM. Statistical analysis: Two-way ANOVA with Sidak´s *post hoc* test. *P ≤ 0.05 and ***P ≤ 0.001 *vs.* control group.

There is a lack of information regarding the effects of Aldo on the subcellular distribution of proteins involved in intracellular Ca^2+^ dynamics. In the immunofluorescence images (**Figure 4**), we observed that Aldo increased the absolute fluorescence of both SERCA isoforms across all subcellular regions of MASMC cells, consistent with their elevated protein expression. Notably, for both isoforms, the subplasmalemmal region exhibited the most pronounced increase. Therefore, we determined the relative expression of both isoforms at each subcellular region of MASMCs. Fluorescence values were normalized to generate a relative distribution; in which each measurement reflects the proportional contribution of a given subcellular region to the total cellular fluorescence. This analysis provided evidence that, in Aldo-treated MASMCs, the subcellular distribution of SERCA2a (**Figure 5A**) and SERCA2b (**Figure 5B**) is remodeled. Data show that the percentual distribution in the subplasmalemmal region increased significantly compared to the control condition. This contrasts with the perinuclear region, where a decrease in the percentual distribution was observed in the Aldo-treated condition compared to the control. No significant changes were observed for both isoforms in the percentual distribution of the cytoplasmic compartment (**Figure 5**). Therefore, in addition to increasing the total protein expression of both SERCA isoforms, Aldo also promotes their expression preferentially in the superficial SR, where SERCA pump contributes to buffering Ca_V_1.2-mediated Ca^2+^ influx through LTCCs.

### 3.6 Aldo increases mitochondrial transcription factors TFAM and TFB2M in mesenteric arteries

Previously, it was reported that TFAM and TFB2M regulate SERCA2 transcription by interacting with the 5’ regulatory region of the SERCA2 gene (Watanabe et al., 2011). Other studies have shown that Aldo regulates the expression of these transcription factors, which directly correlates with changes in SERCA2 expression (Chou et al., 2015). Therefore, before assessing the effects of Aldo on TFAM and TFB2M expression, we confirmed their nuclear localization in MASMC. Three-dimensional (3D) confocal microscopy images of immunolabeled cells with antibodies against TFAM and TFB2M, along with nuclear labeling with DAPI, revealed co-localization of the transcription factors with the nuclear marked, confirming the presence of both proteins in the cell nucleus (**Supplementary Figure 4**).

**Figure 6:**
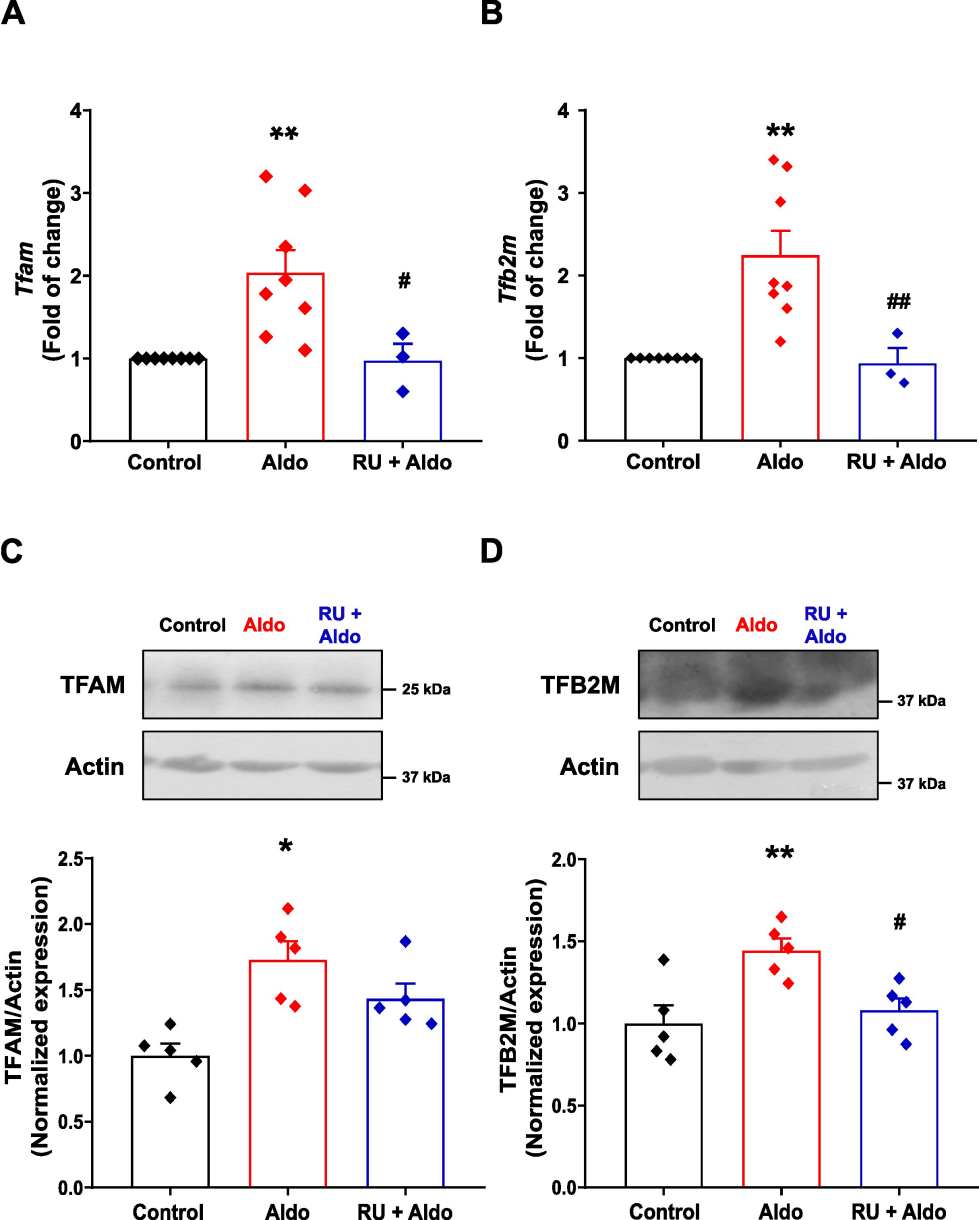
The mitochondrial transcription factors TFAM and TFB2M are increased in rat MA treated with Aldo. **(A, B)**. Scatter plots with bar graphs showing relative mRNA levels of *Tfam* **(A)** and *Tfb2m* **(B)**, determined by real-time qPCR in control (*black symbols/bars*; n = 8 independent experiments/N = 27 rats), Aldosterone-treated (10 nM; *red symbols/bars*; n = 8 independent experiments/N = 27 rats) or co-incubated with RU28328 (1 µM) + Aldo (10 nM, *blue symbols/bars*; n = 3 independent experiments/N = 12 rats) MAs. *Gapdh* was used as reference gene. Data are presented as M ± S.E.M. Statistical analysis: Kruskal-Wallis test followed by Dunn´s multiple comparisons test. **P ≤ 0.01 *vs.* control group. ^#^*P* ≤ 0.05, ^##^*P* ≤ 0.01 *vs*. Aldosterone-treated group. **(C, D)**. Representative immunoblots (*top*) and corresponding normalized scatter plots with bar graphs (*bottom*) showing protein levels of TFAM **(C)** and TFB2M **(D)** in control (*black symbols/bars*; n = 5 independent experiments/N = 20 rats), Aldosterone-treated (10 nM; *red symbols/bars*; n = 5 independent experiments/N = 20 rats) and RU28318 (1 µM) + Aldosterone (10 nM) co-treatment (*blue symbols/bars*; n = 5 independent experiments/N = 20 rats) MAs. Actin was used as loading control. Data are presented as M ± S.E.M. Statistical analysis: One-way ANOVA followed by Tukey *post-hoc* test. *P ≤ 0.05, **P ≤ 0.01 *vs*. control group, ^#^*P* ≤ 0.05 *vs*. Aldosterone-treated group.

Then, we assessed the effect of Aldo on *Tfam* and *Tfb2m*. By using qPCR with specific primers for *Tfam* and *Tfb2m* (**Supplementary Table 3**), we evaluated Aldo effects on the mRNA levels of these two transcription factors. Aldo treatment of MAs significantly increased *Tfam* and *Tfb2m* levels compared with controls, effect that was blocked by the MR antagonist, RU28318 (**Figures 6A, B**), suggesting that Aldo-induced transcriptional effects on *Tfam* and *Tfb2m* are specifically mediated by the MR activation. Once the Aldo transcriptional effects on these mitochondrial factors were evidenced, we analyzed whether the increase in *Tfam* and *Tfb2m* mRNA levels was reflected in their protein expression. Western blot analysis showed that Aldo also increased TFAM (**Figure 6C**) and TFB2M (**Figure 6D**) protein expression, and this effect was also blocked by RU28318. These findings suggest that Aldo promotes SERCA2 gene expression, at least in part, through upregulation of TFAM and TFB2M.

## 4 Discussion

**Figure 7:**
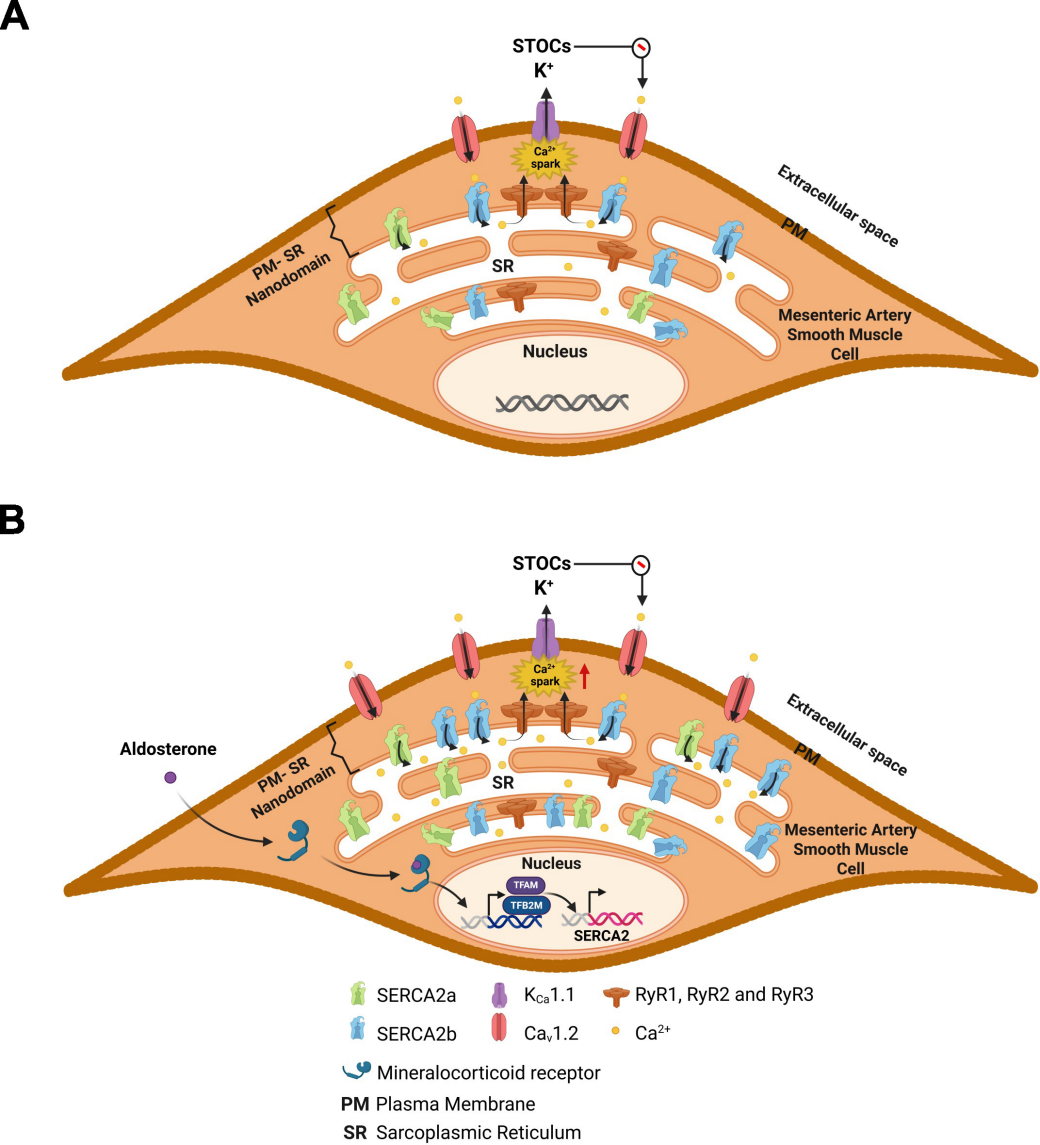
Aldosterone increases the expression of SERCA2a and SERCA2b and enhances their subcellular localization at the superficial SR of mesenteric artery smooth muscle cells. In physiological conditions **(A)** plasma membrane (PM) depolarization induces Ca^2+^ influx through Ca_V_1.2, which is buffered into the SR by the action of the SERCA pump at the PM-SR nanodomain. The increase in SR Ca^2+^ load produces Ca^2+^ sparks by ryanodine receptors (RyR), activating K_Ca_1.1, generating STOCs. The latter decreases the open probability of Ca_V_1.2. Therefore, Ca_V_1.2, SERCA, RyR and K_Ca_1.1 work as a functional unit that regulates intracellular Ca^2+^ dynamics in MASMC. **(B)** Aldo transcriptionally upregulates SERCA2a and SERCA2b expression (via the MR receptor) and increases their localization at the subplasmalemmal region of MASMCs. This SERCA2a and SERCA2b upregulation at the superficial regions is key for buffering the augmented Ca^2+^ influx at the PM-SR nanodomain, which increases Ca^2+^ spark frequency and Ca^2+^ wave incidence in MASMC. The transcriptional effects of Aldo on SERCA2 gene correlate with the augmented expression of TFAM and TFB2M suggesting their involvement in Aldo-mediated enhanced expression of both SERCA2 isoforms.

Our results demonstrate that short-term (24 h) Aldo treatment increased both the frequency of Ca^2+^ sparks and the incidence of Ca^2+^ waves in rat MASMCs (**Figure 1**). These enhanced intracellular Ca^2+^ dynamics are associated with elevated expression of SERCA2a and SERCA2b (**Figures 2**, **4**), accompanied by remodeling of their subcellular distribution (**Figure 5**). Specifically, Aldo promotes the redistribution of SERCA2a and SERCA2b towards the superficial SR (**Figure 5**), likely facilitating more efficient buffering of Ca_V_1.2-mediated Ca^2+^ influx. This redistribution of SERCA2a and SERCA2b may contribute to the increased SR Ca^2+^ load, Ca^2+^ spark frequency and Ca^2+^ wave incidence observed in MASMC exposed to Aldo. The transcriptional effects of Aldo on SERCA2 gene correlate with increased expression of TFAM and TFB2M, suggesting their involvement in Aldo-mediated upregulation of both SERCA2 isoforms (**Figure 6**). Taken together, these findings indicate that the Aldo-induced changes in intracellular Ca^2+^ dynamics are driven by increased SERCA2a and SERCA2b expression and their remodeling within the PM-SR nanodomain (**Figure 7**). Additionally, SERCA2a became enriched in the perinuclear region, where it may sustain SR Ca^2+^ stores that provide Ca^2+^ for contraction or gene transcription. This novel mechanistic perspective helps explain alterations in intracellular Ca^2+^ dynamics in MASMCs following short-term exposure to Aldo, beyond the previously described effects (Salazar-Enciso et al., 2022).

Our previous work demonstrated that Aldo treatment (10 nM, 24 h) increased Ca_V_1.2 expression in rat MA without altering depolarization-induced vasoconstriction or resting [Ca^2+^]_i_, although SR Ca^2+^ content was increased, an effect attributed to SERCA2 upregulation (Salazar-Enciso et al., 2022). In the present study, we investigate whether this Aldo-mediated SERCA upregulation involved only SERCA2b, the predominant isoform in VSMCs, or both major isoforms, SERCA2a and SERCA2b. We also examined the impact of Aldo on the subcellular distribution of both SERCA isoforms, a novel perspective that may explain changes in intracellular Ca^2+^ dynamics in MASMCs.

Our results showed that Aldo-treated MASMCs exhibit a significant increase in Ca^2+^ spark frequency, as well as a higher proportion of cells exhibiting Ca^2+^ waves (**Figure 1**). These findings correlate with the elevated SR Ca^2+^ load induced by Aldo in rat MASMCs, previously reported by our group (Salazar-Enciso et al., 2022), consistent with the well-established dependence of local Ca^2+^ signal initiation on SR Ca^2+^ content (ZhuGe et al., 1999; Cheranov and Jaggar, 2002; Fan et al., 2018), and SERCA activity (Takeda et al., 2011).

Interestingly, the Ca^2+^ spark decay-time, a parameter linked to cytoplasmic Ca^2+^ clearance mechanisms, was significantly decreased in MASMC exposed to Aldo (**Figure 1**), suggesting an important contribution of the SERCA pump in the local Ca^2+^ decline of the Ca^2+^ spark; however, we cannot rule out other factors determining the decay of Ca^2+^ sparks, for instance, Ca^2+^ diffusion, Na^+^/Ca^2+^ exchanger activity, and cytoplasmic proteins buffering Ca^2+^ (sorcin, calmodulin, etcetera) than may participate in the effects of Aldo in MASMC (Jaggar, 2001; Cheranov and Jaggar, 2002; Rueda et al., 2006). These factors may participate together with SERCA in mediating Aldo effects in the Ca^2+^ spark decay-time in MASMCs.

The SR Ca^2+^ load is regulated by SERCA pump activity, an enzyme located in the SR membrane that recaptures cytosolic Ca^2+^ into the SR (Clark et al., 2010; Takeda et al., 2011; Van Breemen et al., 2013; Evans, 2017). Hormones such as progesterone have been shown to increase SERCA2 expression, thereby augmenting SR Ca^2+^ load (Moshal et al., 2014; Pang and Thomas, 2021). In contrast, the effects of Aldo on SERCA expression remained poorly understood. Rat MAs express both SERCA2a and SERCA2b, and in the present study we provide evidence that short-term (24 h) Aldo exposure upregulates both SERCA isoforms at the transcriptional level, as reflected by increased mRNA levels. The regulation of SERCA by Aldo represents a virtually unexplored field; thus, it is critical to elucidate how this hormone reshapes SERCA activity, given the essential role of this pump in the ignition of local Ca^2+^ signals which participate in vaso-relaxation or vasoconstriction. Only a few studies have examined this relationship. Chou et al. (2015) reported that Aldo decreased SERCA2 expression in primary cultures of HAOSMCs, after 48 h of exposure to 10 nM or 100 nM Aldo (Chou et al., 2015). Discrepancies between their findings and ours may be explained by different experimental conditions, vascular beds, or species. HAOSMCs cultured in growth medium undergo a phenotypic switch to a proliferative phenotype (Berra-Romani et al., 2008; Chou et al., 2015), whereas our model preserves the contractile phenotype. Bobe et al. (2010) demonstrated that the transition from a contractile to a proliferative phenotype decreases SERCA2a expression in human carotid arteries (Bobe et al., 2010). Therefore, the effects of Aldo on SERCA may be dependent on the phenotypic state. Importantly, we confirm that the changes observed in our experimental model were not attributable to phenotypic modifications. Beyond this, the precise explanation for the differences between studies remains unclear.

In VSMCs, the SR is functionally segregated, and evidence suggests that this segregation creates distinct functional Ca^2+^ stores that are mobilized in response to specific stimuli (Clark et al., 2010; Van Breemen et al., 2013). In pulmonary artery VSMCs, the differential subcellular localization of SERCA2a and SERCA2b (the predominant isoforms) appears to contribute to the generation of different intracellular Ca^2+^ signals (Clark et al., 2010). Approximately 90% of SERCA2a is concentrated in the perinuclear region, whereas SERCA2b is mainly expressed in the subplasmalemmal region, with about 72% localized to the PM-SR nanodomain (Clark et al., 2010).

Our results show that SERCA2a is primarily found in the perinuclear region of MASMCs. In contrast, SERCA2b is distributed almost equally between the subplasmalemmal and perinuclear regions, with both isoforms expressed at lower levels in the cytoplasmic (or extraperinuclear) region. These findings agree with the subcellular distribution of both SERCA isoforms in pulmonary artery VSMCs (Clark et al., 2010). Our results are also in agreement with previous findings in guinea pig urinary bladder smooth muscle cell, showing SERCA pump largely confined to the perinuclear and subplasmalemmal regions (Gómez-Viquez et al., 2010).

Regarding the effects of Aldo on SERCA isoform subcellular localization, we found that exposure to the mineralocorticoid increases the expression of SERCA2a and SERCA2b in all subcellular regions. However, this hormone appears to initiate a remodeling in the distribution of both isoforms, since after analyzing the percentage of expression in all regions and comparing it with that obtained in the control condition, we found that the percentage of expression of both isoforms is increasing in the subplasmalemmal region, while in contrast, the percentage of expression is decreasing in the perinuclear region. These regionally selective changes in SERCA pump distribution are also promoted by other molecular effectors, such as thyroid hormones and glucose (Arruda et al., 2005; Searls et al., 2010). In skeletal muscle of hyperthyroid animals, SERCA1 and SERCA2 pump expression changes specifically in one subcellular region: SERCA1 increases while SERCA2 decreases compared to control animals. This leads to an increased rate of Ca^2+^ uptake in that subcellular region (Arruda et al., 2005). In VSMC of aortas obtained from animal models of induced diabetes, changes in SERCA pump expression were observed, with differential increases shifting from a generalized distribution to a more perinuclear location, also with changes in Ca^2+^ signaling (Searls et al., 2010). This work also shows that when A7r5 cells, a cellular line derived from rat thoracic aorta, were exposed to high glucose concentrations, SERCA2 expression increased, specifically in the perinuclear SR region. The mechanism underlying these effects is unknown, but it is important to highlight the significant role of the SERCA pump in these pathological phenomena (Searls et al., 2010).

The increase in SERCA2a and SERCA2b expression correlates with our previous findings of increased SR Ca^2+^ load in Aldo-treated MASMC (Salazar-Enciso et al., 2022). The augmented SR Ca^2+^ content modifies the frequency of Ca^2+^ sparks and the incidence of Ca^2+^ waves (Lee et al., 2005; Cheng and Lederer, 2008), suggesting an efficient buffering of Ca^2+^ influx at the superficial regions of VSMCs where the PM-SR nanodomain is formed. In VSMCs, the superficial SR acts as a dynamic Ca^2+^ store that buffers Ca^2+^ entry, also known as the superficial buffer barrier (Van Breemen et al., 1995, Van Breemen et al., 2013). The transient retention of Ca^2+^ in the superficial SR reduces its diffusion and affects the temporal and spatial organization of the Ca^2+^ signal generated by Ca^2+^entry through the PM. This is related to the fact that contraction is more associated with the rate of Ca^2+^ influx than with the magnitude of incoming Ca^2+^ that is intercepted by the superficial SR before reaching the deep cytoplasm (Van Breemen, 1977; Van Breemen et al., 2013). A physiologically regulated superficial buffer barrier allows VSMC to utilize Ca^2+^ as a highly structured and spatially compartmentalized signal, rather than a simple global trigger for contraction, thus enabling gradual modulation of tone (Van Breemen, 1977; Van Breemen et al., 1995, Van Breemen et al., 2013). This compartmentalization of the Ca^2+^ signal allows continuous variations in influx dynamics to translate into progressive changes in the contractile response (Van Breemen, 1977). Therefore, pharmacological manipulation of Ca^2+^ transport in the superficial SR offers an alternative pathway for controlling vascular tone without directly blocking Ca^2+^ influx, by modifying how the Ca^2+^ signal is processed within the cell rather than the total amount of Ca^2+^ that crosses the PM. In this context, the Aldo-induced subplasmalemmal increase in SERCA2a and SERCA2b suggest a functional buffering mechanism whereby, even in the presence of a greater influx of Ca^2+^ is efficiently taken up by the superficial SR our cells, limiting diffusion into deep cytoplasm. This mechanism provides an explanatory framework for the functional observations previously reported by our research group, in which neither the depolarization-induced contraction nor the resting [Ca^2+^]_i_ showed differences compared to the controls (Salazar-Enciso et al., 2022). Our data indicate that short-term exposure to Aldo establishes a new steady state of intracellular Ca^2+^ cycling at resting/basal conditions. In this state, SERCA2a/2b upregulation and strategic localization at PM-SR nanodomains counterbalance unsought elevations of [Ca^2+^]_i_. However, the concomitant increase in luminal SR Ca^2+^ levels may also participate in enhancing receptor-mediated Ca^2+^ releases, thereby promoting abnormal vasoconstriction, a hallmark feature of overactive Aldo/MR signaling in models of chronic exposure to Aldo. We acknowledge that the net functional outcome has not been explored in sufficient detail, and we plan to investigate this aspect in future studies.

Aldo plays a central role in vascular remodeling, fluid and electrolyte homeostasis, and blood pressure regulation (Lother et al., 2015). Clinical studies have evidenced that the incidence of hypertension increases with elevated serum Aldo levels. Moreover, primary hyperaldosteronism has been identified as a major etiological factor in secondary and resistant hypertension, conditions strongly associated with increased cardiovascular morbidity, stroke, and acute myocardial infarction (Xanthakis and Vasan, 2013; Monticone et al., 2017, Monticone et al., 2018). Therefore, it is reasonable to consider that chronic exposure to pathological Aldo levels contributes to vascular damage, enhanced vasoconstriction, impaired vaso-relaxation, and the development of hypertension. In fact, alterations in the expression of key Ca^2+^ handling proteins, including the SERCA pump, have been reported in hypertension models (Zulian et al., 2010; Hadri et al., 2013). However, the specific actions of Aldo actions Ca^2+^ handling proteins of the PM-SR nanodomain remain elusive in VSMCs under acute, short-term, or long-term exposure. In the present study, we demonstrate that early genomic effects of Aldo involve upregulation and subcellular redistribution of SERCA2a and SERCA2b. We think that these short-term effects are compensatory, counteracting immediate disruptions in Ca^2+^ homeostasis. We further hypothesize based on previous experience, that increased SERCA pump expression cannot be sustained for prolonged periods of time, as observed in chronic disease conditions (Miklós et al., 2012; Vazquez-Jimenez et al., 2016; Romero-García et al., 2025); accordingly, chronic exposure to supraphysiological Aldo levels may ultimately downregulate SERCA expression. Although our *ex vivo* model does not mimic chronic conditions, it introduces the SERCA pump as a novel target of Aldo-mediated vascular remodeling at early stages.

TFAM and TFB2M are essential components of the mitochondrial transcription machinery (Ramachandran et al., 2017). TFAM binds promoters’ sequences of mitochondrial DNA and facilitates the recruitment of mitochondrial RNA polymerase. TFAM also contributes to the structural organization of mtDNA into nucleoids (Lee et al., 2014; Ramachandran et al., 2017; Bonekamp et al., 2021). TFB2M acts as an initiation factor for transcription by interacting with the RNA polymerase inducing the local opening of DNA at promoters’ sites, thus enabling the initiation of mitochondrial RNA synthesis (Ramachandran et al., 2017). While their primary function is in the mitochondria, these proteins have been reported in the nucleus of some cell types (Watanabe et al., 2011; Lee et al., 2014; Lionaki et al., 2016). TFAM has been detected in the nuclei of neuronal cells in the hippocampus (Lee et al., 2014). TFAM and TFB2M have also been observed in the nuclei of neonatal rat cardiomyocytes, where they interact with nuclear chromatin (Watanabe et al., 2011). Furthermore, two nuclear localization signals have been identified within the protein domains of TFAM (Pastukh et al., 2007; Lionaki et al., 2016), arguing in favor of a key role of these two mitochondrial factors in the transcription of nuclear DNA genes.

Specifically, SERCA2 gene expression is positively regulated by TFAM and TFB2M, which interact directly with the 5’ regulatory region of the promoter. These interactions suggest that SERCA2 expression is tightly coupled to transcriptional mechanisms dependent on factors associated with mitochondrial function (Watanabe et al., 2011). Aldo modifies the expression of these two transcription factors, and changes in SERCA2 expression induced by this hormone have been described in which TFAM and TFB2M are part of a mechanism regulating SERCA2 pump expression (Chou et al., 2015). In this context, Aldo´s modulation of these transcription factors represents a potential point of convergence between hormonal signaling and the control of Ca^2+^ homeostasis.

Exploring this potential mechanism, we found that both factors are upregulated by Aldo, which may directly account for the observed increase in SERCA2a and SERCA2b expression. This increase suggests that Aldo-induced changes in SERCA2 expression do not occur in isolation, but rather as part of a broader transcriptional mechanism that regulates key factors associated with energy control and indirectly intracellular Ca^2+^ handling In this regard, the upregulation of TFAM and TFB2M could contribute to maintaining a greater Ca^2+^ reuptake capacity, thus promoting functional adjustments in MASMCs. In the strict sense of modulation, our results are consistent with those reported in the literature on the expression of TFAM and TFB2M under the effects of Aldo, reinforcing the idea that this hormone can indirectly influence SERCA2 expression through the regulation of these transcription factors (Chou et al., 2015). However, the discrepancies observed with the effects described by Chou et al., both the expression of SERCA2 and these transcription factors, could be related to differences in the phenotypic state of the cells analyzed (Chou et al., 2015). It is well known that VSMCs exhibit remarkable phenotypic plasticity (Liu et al., 2022).

Mitochondrial metabolism is a key determinant of phenotypic transition in VSMCs (Oller et al., 2021; Liu et al., 2022). Alterations in mitochondrial function are associated with profound changes in cell identity, affecting both the expression of contractile proteins and the ability to handle Ca^2+^ (Berra-Romani et al., 2008; Oller et al., 2021; Liu et al., 2022). Either deletion or decreased TFAM expression has been associated with mitochondrial dysfunction, a condition also associated with the transition of the contractile phenotype of VSMCs towards a synthetic or proliferative state (Oller et al., 2021).

Taken together, our results demonstrated that Aldo is a key regulator of intracellular Ca^2+^ dynamics in MASMCs under pathological concentrations. Aldo consistently increases the incidence of Ca^2+^ sparks and Ca^2+^ waves in the PM-SR nanodomain, in association with increased expression of SERCA2a and SERCA2b and remodeling of their subcellular localization toward the subplasmalemmal region. This distribution establishes a highly efficient superficial buffering barrier against Ca^2+^ influx, increasing the Ca^2+^ load in the SR and promoting the generation of local Ca^2+^ signals. In this context, the Aldo-induced increase in TFAM and TFB2M expression emerges as a component in the regulation of SERCA2 expression.

### 4.1 Limitations of the study

We acknowledge several limitations in our study. First, the 24-hour incubation period with Aldo, while sufficient to assess short-term adaptive responses, may not fully capture the long-term effects of Aldo exposure. Second, vascular tone was not directly measured, which limits our ability to establish a functional link between the observed molecular changes and the contractile response of the arteries. In this regard, complementary myographic assays would therefore provide a more precise evaluation of the overall functional impact of Aldo on MAs. Third, although TFAM and TFB2M binding sites are predicted in the SERCA2 gene promoter, their presence in the vasculature was not directly demonstrated. This limits mechanistic confirmation of a SERCA transcriptional regulation mediated by these factors in MA. Finally, we cannot yet distinguish the precise contribution of each SERCA isoform to the observed increase in intracellular Ca^2+^ dynamics, whether in the form of sparks or waves. Given SERCA2b is the predominant isoform in rat MA, we hypothesize that this pump plays a major in increasing Ca^2+^ spark frequency and Ca^2+^ wave incidence. This hypothesis, however, requires direct experimental validation. Future studies integrating functional assays with specific molecular approaches will be essential to strengthen and refine the interpretation of the findings of this work.

## Data Availability

The raw data supporting the conclusions of this article will be made available by the authors, without undue reservation.
