# Supplementary material for: Aldosterone increases the expression and subcellular localization of SERCA2a and SERCA2b in the rat mesenteric artery
Source: Front Physiol. 2026 May 4;17:1811001. doi: 10.3389/fphys.2026.1811001 (PMC13180557; doi:10.3389/fphys.2026.1811001)
Supplement: Supplementary file 1 [file Table1.docx]

***Supplementary Material***

Arriero-Carrillo et al., 2026


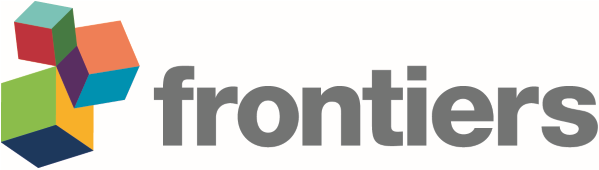


## Supplementary Tables

**Supplementary Table 1. List of reagents and resources by type of experiment**

| **Reagent** | **Source** | **Catalog number** |
| --- | --- | --- |
| **Mesenteric artery *ex-vivo* incubation** | | |
| DMEM | Invitrogen | 11885084 |
| Aldosterone | Sigma-Aldrich | A9477 |
| RU28318 | Tocris Bioscience | 1672 |
| Penicillin-Streptomycin | GIBCO | 15140122 |
| **Mesenteric artery smooth muscle cell isolation** | | |
| Collagenase F | Sigma-Aldrich | C7926 |
| Collagenase H | Sigma-Aldrich | C8051 |
| Papain | Sigma-Aldrich | P4762 |
| BSA | Sigma-Aldrich | A8806 |
| DTT | Sigma-Aldrich | D-0632 |
| **Immunostainings** | | |
| Sheep serum | Biowest | BIO-S2350-500 |
| Circular coverslip #1, 1 oz, 18 mm | Daiggerbrand | 12-545-100 |
| Slowfade diamond antifade mountant with DAPI | Molecular probes | S36973 |
| **Western blot** | | |
| PVDF membranes | MilliporeSigma | IPV00010 |
| SuperSignal west Pico | Thermo Fisher Scientific | 34080 |
| SuperSignal west Femto | Thermo Fisher Scientific | 34096 |
| Acrylamide | Sigma-Aldrich | A8887 |
| Bicinchoninic acid (BCA) method | Pierce | 23225 |
| **Real-time qPCR** | | |
| Tri Reagent^®^ | Sigma-Aldrich | T9424 |
| Super Script II Transcriptase Kit | Invitrogen | 18064-014 |
| Rotor-Gene SYBR Green PCR Kit | Qiagen | 204074 |
| **Recording of Ca^2+^ sparks and Ca^2+^ waves** | | |
| Fluo 4-AM | Invitrogen | F14201 |
| Cyclopiazonic acid | Sigma | C1530 |

**Supplementary Table 2. Antibodies**

**Western blot:**

| **Target** | **Source** | **Catalog number** | **Dilution** | **Secondary Ab** |
| --- | --- | --- | --- | --- |
| SERCA2a | Badrilla | A010-23S | 1:20,000 | Anti rabbit (1:5,000) |
| SERCA2b | Badrilla | A010-24S | 1:5,000 | Anti rabbit (1:5,000) |
| TFAM | Santa Cruz Biotechnology | sc-166965 | 1:500 | Anti mouse (1:5,000) |
| TFB2M | Santa Cruz Biotechnology | sc-517095 | 1:500 | Anti mouse (1:5,000) |
| GAPDH | Ambion | AMA4300 | 1:50,000 | Anti mouse (1:5,000) |
| Actin | Sigma-Aldrich | A5060 | 1:20,000 | Anti rabbit (1:5,000) |

**Immunostainings:**

| **Target** | **Source** | **Catalog number** | **Dilution** |
| --- | --- | --- | --- |
| SERCA2a | Badrilla | A010-23S | 1:200 |
| SERCA2b | Badrilla | A010-24S | 1:200 |
| TFAM | Santa Cruz Biotechnology | sc-166965 | 1:100 |
| TFB2M | Santa Cruz Biotechnology | sc-517095 | 1:100 |
| Alexa Fluor™ 488 Goat Anti-Rabbit IgG | Invitrogen | A31627 | 1:400 |
| Alexa Fluor™ 488 Goat Anti-Mouse IgG | Invitrogen | A11017 | 1:400 |

Ab, antibody; GAPDH, Glyceraldehyde-3-phosphate dehydrogenase; SERCA2a, sarcoplasmic/endoplasmic reticulum calcium ATPase 2 isoform 2a; SERCA2b, sarcoplasmic/endoplasmic reticulum calcium ATPase 2 isoform 2b; TFAM, Mitochondrial transcription factor A; TFB2M, Mitochondrial transcription factor B2.

**Supplementary Table 3. Real-time qPCR primers**

| Target Gen | Sequence accession number | Primer | Sequence | Fragment (pb) |
| --- | --- | --- | --- | --- |
| *Atp2a2a* | NM_001110139.2 | Forward | 5’-GGCTGATGGTGCTGAAAATCTC-3’ | 115 |
|  |  | Reverse | 5’-CAATGTTTAGGAAGCGGTTACTCC-3’ |  |
| *Atp2a2b* | NM_001110823.2 | Forward | 5’-AAACCCTCCTGCTCCCTGTC-3’ | 90 |
|  |  | Reverse | 5’-GCTGTAGACCCAGACCACCAG-3’ |  |
| *Tfam* | NM_031326.2 | Forward | 5’-GTCTCATGATGAAAAGCAGGCAT-3’ | 121 |
|  |  | Reverse | 5’- ACGGATGAGATCACTTCGCC -3’ |  |
| *Tfb2m* | NM_001008293.1 | Forward | 5’- ACGGATGAGATCACTTCGCC -3’ | 96 |
|  |  | Reverse | 5’- CGTGTCTCCAGGTCTTTTCCTT -3’ |  |
| *Gapdh* | NM_017008.4 | Forward | 5’-CTGCACCACCAACTGCTTAG-3’ | 92 |
|  |  | Reverse | 5´-TGATGGCATGGACTGTGG-3’ |  |
| *Tagln* | NM_031549.2 | Forward | 5’-GTTTGGCCGTGACCAAGAAC-3’ | 129 |
|  |  | Reverse | 5’-GAAGGCCAATGACGTGCTTC-3’ |  |

*Atp2a2a,* sarcoplasmic/endoplasmic reticulum calcium ATPase 2 isoform 2a; *Atp2a2b,* sarcoplasmic/endoplasmic reticulum calcium ATPase 2 isoform 2b; *Gapdh,* Glyceraldehyde-3-phosphate dehydrogenase; *Tagln,* Transgelin; *Tfam,* Mitochondrial transcription factor A; *Tfb2m,* Mitochondrial transcription factor B2; pb, pair bases. Information obtained from MFE primer 3.1 (Wang et al., 2019)

## Supplementary Figures


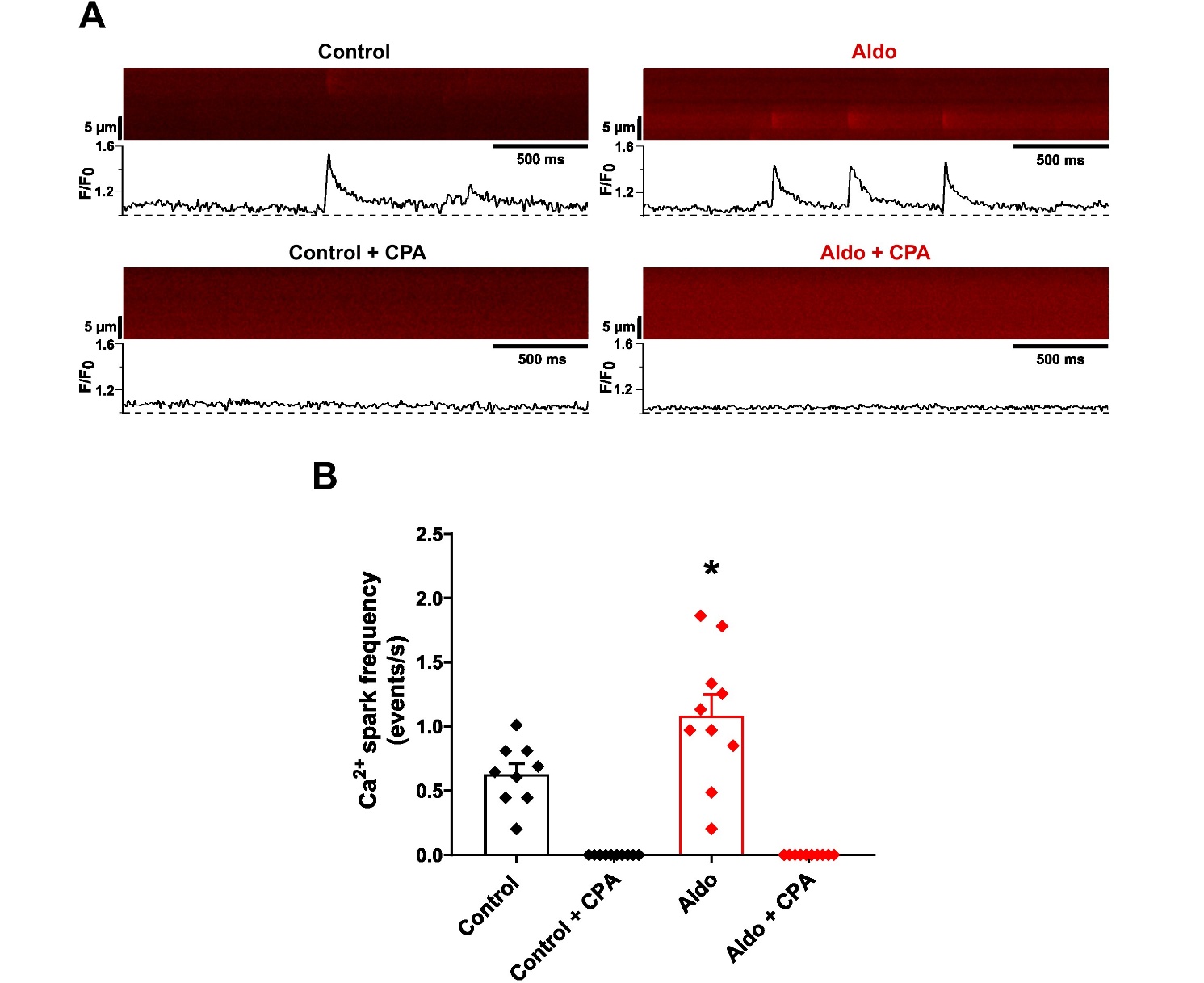


**Supplementary Figure S1. Cyclopiazonic acid suppresses Ca^2+^ spark activity in control and Aldo-treated MASMCs**. **A.** Representative pseudo-colored confocal images of Ca^2+^ sparks and normalized fluorescence profiles (F/F_0_) from individual MASMCs recorded in Fluo 4-loaded MAs in the absence (Control, *left*) or the presence of Aldo (Aldo 10 nM, 24 h, *right*). Ca²⁺ sparks were recorded as described in Materials and Methods section. Fluo 4-loaded MAs were superfused with PSS-20K alone to record Ca^2+^ sparks in basal condition (*top images and traces*); then the same MAs were superfused with PSS-20K plus cyclopiazonic acid for 10 min (CPA 10 µM). After the 10-min incubation, the protocol for recording Ca^2+^ sparks was applied (*bottom images and traces*). **B**. Scatter plot with bar graph of Ca^2+^ spark frequency (determined as the number of Ca^2+^ events per s). Data are shown for Control (*black symbols*/*bars*, 167 sparks, n = 10 cells) and Aldo-treated MASMC (*red symbols*/*bars*, 240 sparks, n = 10 cells) in basal conditions. The incubation with CPA for 10 min blocked Ca^2+^ spark activity. Values are presented as M ± S.E.M. The Mann-Whitney U test was used to determine significant differences; **P* ≤ 0.05 *vs*. control group.


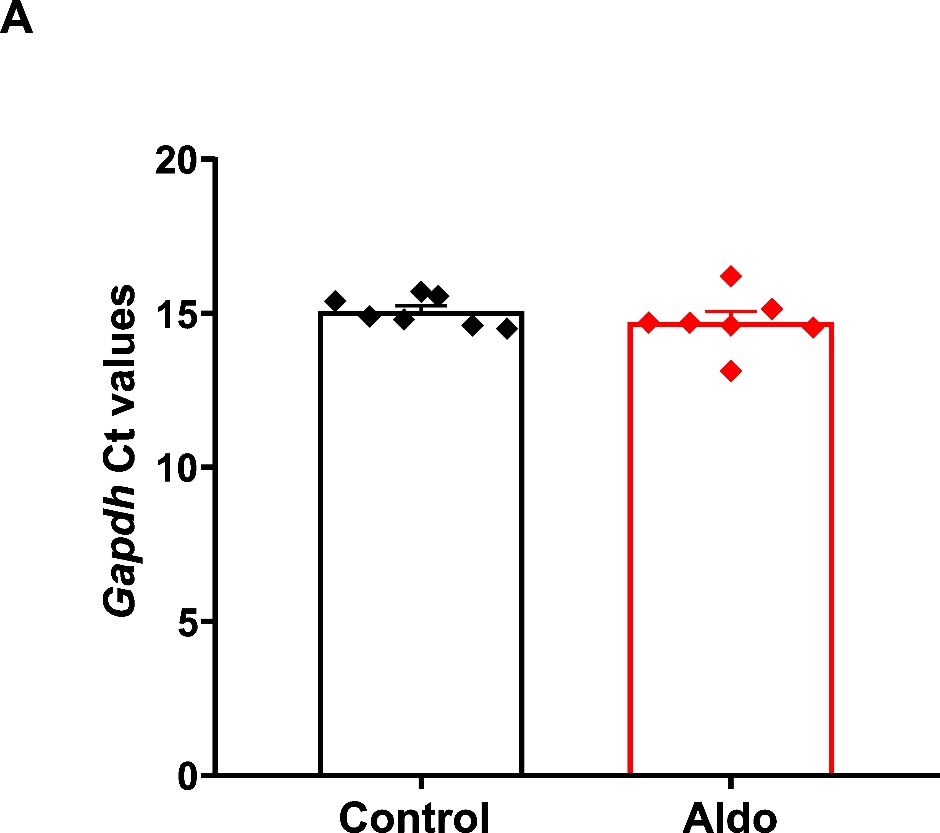


**Supplementary Figure S2. *Gapdh* as a reliable reference gene for real time qPCR. A.** Scatter plot with bar graph of the threshold cycle (Ct) for *Gapdh* determined by real time qPCR in control (*black symbols/bar*, n=7 independent experiments) and Aldosterone-treated (10 nM; *red symbols/bar*, n = 7 independent experiments/21 rats) MA. Each sample was prepared with three pooled MA segments, as reported previously (Salazar-Enciso et al., 2022). Data are presented as M ± SEM. Statistical analysis: Unpaired Student t-test.

**
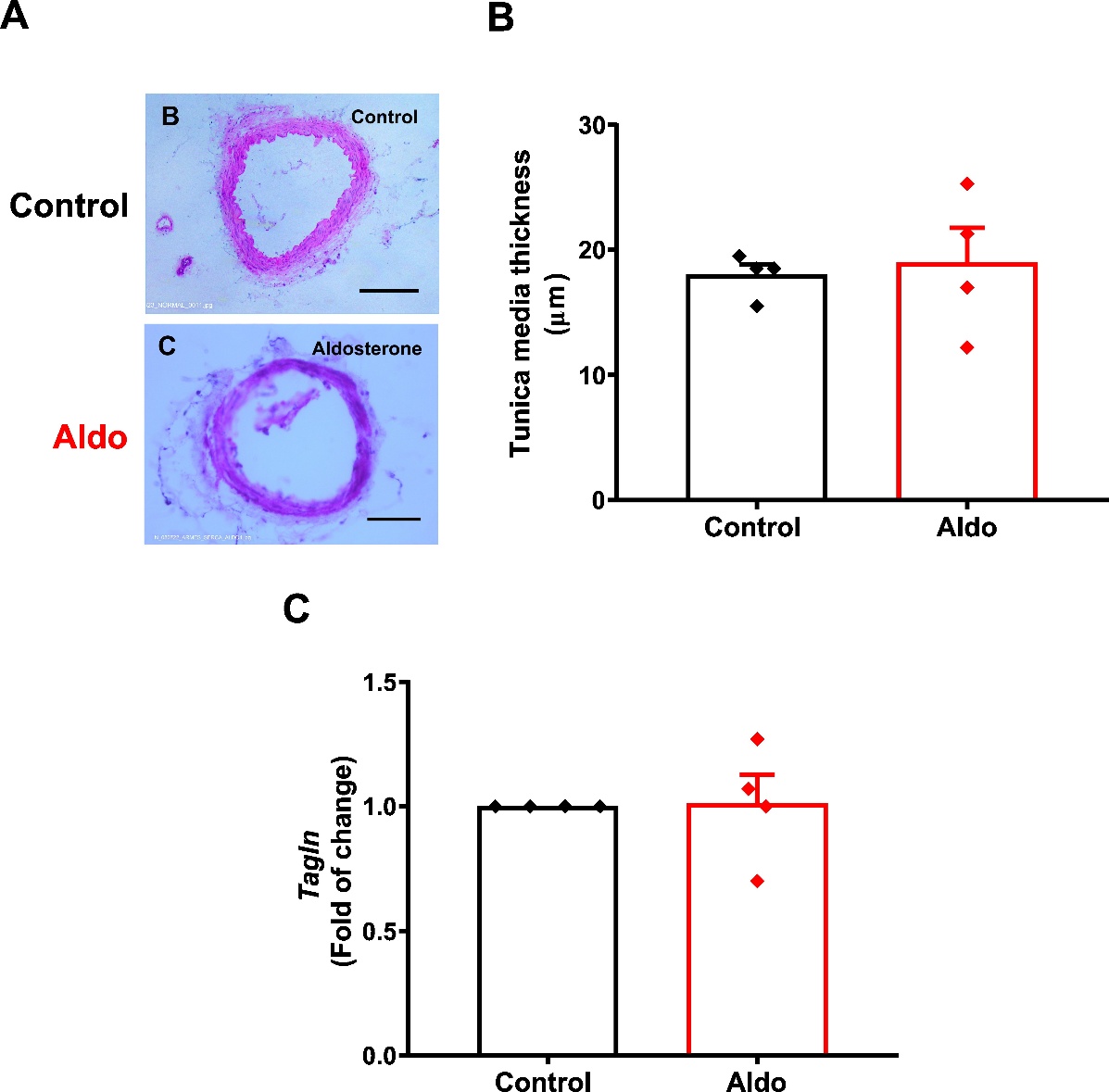
**

**Supplementary Figure S3. Exposure to Aldo for 24 hours does not change the phenotype of MASMCs. A.** Representative transverse (cross-sectional) images of control (*top*) and Aldo-treated MA (*bottom*) obtained by optical microscopy. Segments of resistance-sized MA were sectioned at 10 µm-thick on a cryostat microtome and stained with hematoxylin and eosin, are described below (Supplementary Material and Methods). Scale bar = 50 µm. **B.** Scatter plot with bar graph showing tunica media thickness of segments of MA treated with Aldo (*red symbols/bar,* n = 4 experiments/4 rats) and their respective control (*black symbols/bar*, n = 4 experiments/4 rats). Data are presented as the M ± SEM. Unpaired Student t-test. **C.** Scatter plot with bar graph of relative mRNA levels of transgelin (*Tagln*) also known as smooth muscle protein 22-alpha (SM22-alpha) determined by real-time qPCR in control (*black symbols/bar*, n = 4 experiments/12 rats), and Aldosterone-treated (10 nM; *red symbols/bar*, n = 4 experiments/12 rats). Data are presented as M ± SEM. Statistical analysis: Unpaired Student t-test.


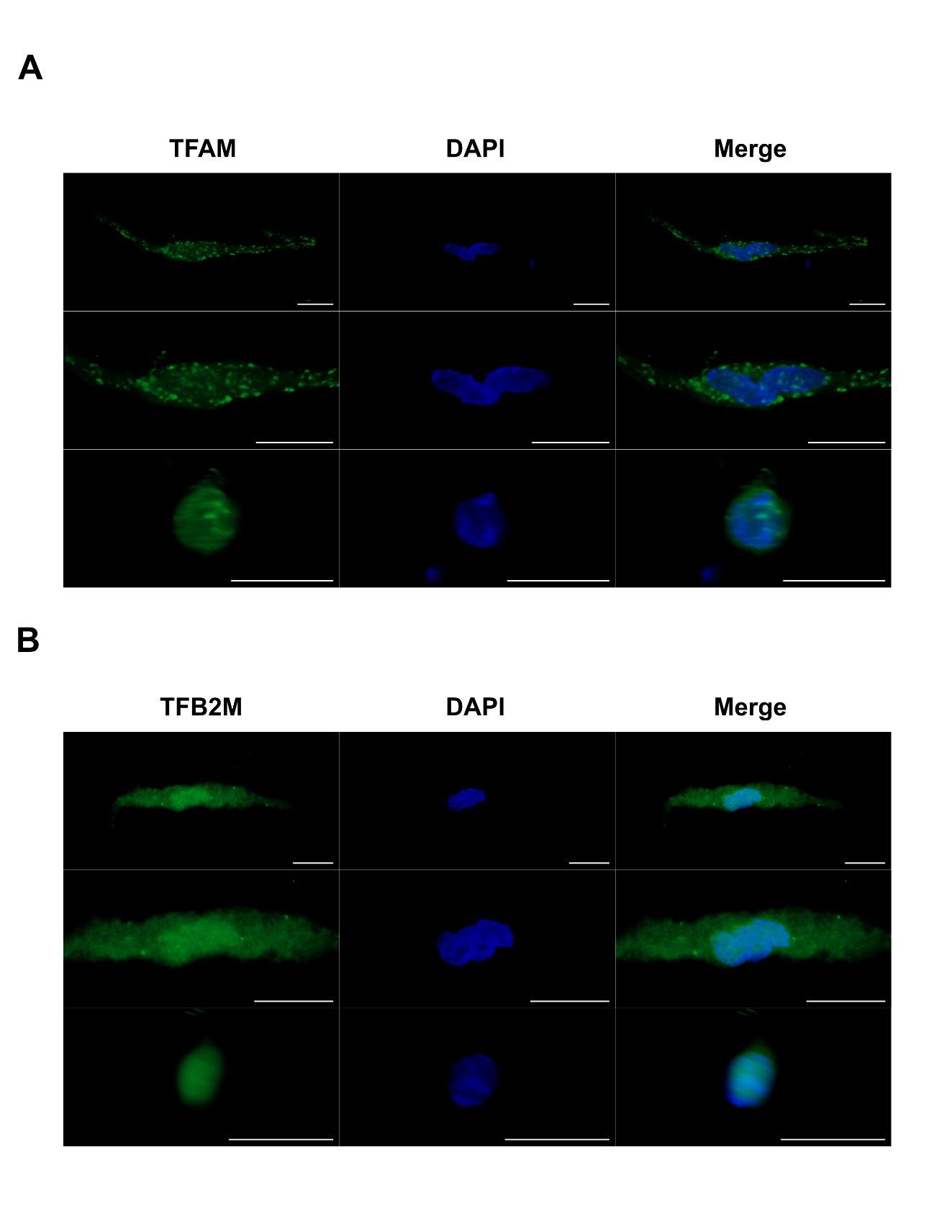


**Supplementary Figure S4. Subcellular localization of TFAM and TFB2M in MASMCs.** Representative 3D fluorescence images obtained by confocal microscopy of single MASMC immunostained with antibodies against mitochondrial transcription factor TFAM (**A**) or TFB2M (**B**). 3D reconstructions showing the whole cell (*top*), a close-up of the nuclear region (*middle*), and a cross-sectional image of the nuclear region in the xz-plane (*bottom*). The images correspond to representative z-projections of the fluorescent signal. The transcription factors TFAM and TFB2M are shown in *green*, cell nucleus in *blue*, and the colocalization pixels in the Merge image in *cyan*. Scale bar = 10 µm.

**Supplementary Materials and Methods**

**Immunohistochemistry**

After the incubation period with Aldo (24 h) rat MA segments were collected and fixed in freshly prepared 3.5% (w/v) formaldehyde in PBS for 2 h. Following fixation, tissues were dehydrated and embedded in paraffin. Serial sections of 8 μm thickness were cut and stained with hematoxylin–eosin (H&E)(Humason, 1972). Sections were mounted on slides using neutral mounting medium D.P.X. (Sigma-Aldrich, 317616) and examined under a Leica DM2000 microscope (Leica Microsystems GmbH, Wetzlar, Germany). Arteries were observed with a 40× objective, and representative images were captured using a Leica DFC425C digital camera (Leica Microsystems GmbH, Wetzlar, Germany). Measurements of the tunica media were performed with ImageJ software (version 1.54m; Rasband, W.S., National Institutes of Health, Bethesda, Maryland, USA; <https://imagej.net/ij/>), calibrated against a micrometer scale. Each arterial section was measured at 10 distinct positions to minimize variability associated with histological sectioning. Results represent the average of three fields from three different rats for each experimental condition.

**Immunocytochemistry of the transcription factors TFAM and TFB2M**

Immunocytochemistry was performed as described in section 2.6 of Materials and Methods, with some modifications. Fixed MASMCs were incubated overnight at 4 °C with primary antibodies against the mitochondrial transcription factor A (TFAM, 1:100 in PBS-T) or B2 (TFB2M, 1:100 in PBS-T). After the incubation with the primary antibodies, MASMCs were washed three times with PBS and incubated with the secondary antibody, Alexa-Fluor 488 goat anti-mouse IgG (1:400 in PBS-T, Invitrogen Cat# A11017) for 2 h at RT in the dark, then washed three times with PBS. Coverslips were mounted on slides with a mounting solution containing DAPI. Images were acquired with a Zeiss LSM 900 confocal microscope using Zen 210 software (Carl Zeiss de México S.A. de C.V.). Stacks of cell images were acquired by optically sectioning cells every 0.3 µm in the z-plane with a Zeiss Apochromatic 63x oil immersion objective; the pinhole was optimized to 1 Airy unit (pixel size 0.071 µm x 0.071 µm x 0.3 µm). Cell images were collected by alternating the excitation of Alexa Fluor 488 with the solid-state 488 laser line, and DAPI with the solid-state 405 laser line. Both, three-dimensional (3D) reconstructions and image analyses were performed with Image J/Fiji software (v.1.54p, National Institutes of Health, USA).

**References**

Humason, G. L. (1972). “Specific staining methods,” in *Animal tissue techniques*, eds. D. Kennedy and RB. Park (San Francisco: W.H. Freeman), 183–185.

Salazar-Enciso, R., Guerrero-Hernández, A., Gómez, A. M., Benitah, J. P., and Rueda, A. (2022). Aldosterone-Induced Sarco/Endoplasmic Reticulum Ca2+ Pump Upregulation Counterbalances Cav1.2-Mediated Ca2+ Influx in Mesenteric Arteries. *Front. Physiol.* 13, 834220. doi: 10.3389/fphys.2022.834220

Wang, K., Li, H., Xu, Y., Shao, Q., Yi, J., Wang, R., et al. (2019). MFEprimer-3.0: Quality control for PCR primers. *Nucleic Acids Res.* 47, W610–W613. doi: 10.1093/nar/gkz351
